# Nanotherapeutics with immunoregulatory functions for the treatment of bacterial infection

**DOI:** 10.1186/s40824-023-00405-7

**Published:** 2023-07-22

**Authors:** Dongliang Yang, Meng Ding, Yanni Song, Yanling Hu, Weijun Xiu, Lihui Yuwen, Yannan Xie, Yingnan Song, Jinjun Shao, Xuejiao Song, Heng Dong

**Affiliations:** 1 Key Laboratory of Flexible Electronics (KLOFE) and Institute of Advanced Materials (IAM), School of Physical and Mathematical Sciences, Nanjing Tech University (NanjingTech), Nanjing, 211816 China; 2 Nanjing Stomatological Hospital, Affiliated Hospital of Medical School, Nanjing University, Nanjing, 210008 China; 3 State Key Laboratory of Organic Electronics and Information Displays & Institute of Advanced Materials (IAM), Nanjing University of Posts & Telecommunications, 9 Wenyuan Road, Nanjing, 210023 China; 4 Department of Physiology, School of Basic Medical Sciences, Guizhou Medical University, Guiyang, 550025 China

**Keywords:** Immune regulation, Immunotherapy, Nanotherapeutics, Bacterial infection, Combined therapy

## Abstract

The advent of drug-resistant pathogens results in the occurrence of stubborn bacterial infections that cannot be treated with traditional antibiotics. Antibacterial immunotherapy by reviving or activating the body’s immune system to eliminate pathogenic bacteria has confirmed promising therapeutic strategies in controlling bacterial infections. Subsequent studies found that antimicrobial immunotherapy has its own benefits and limitations, such as avoiding recurrence of infection and autoimmunity-induced side effects. Current studies indicate that the various antibacterial therapeutic strategies inducing immune regulation can achieve superior therapeutic efficacy compared with monotherapy alone. Therefore, summarizing the recent advances in nanomedicine with immunomodulatory functions for combating bacterial infections is necessary. Herein, we briefly introduce the crisis caused by drug-resistant bacteria and the opportunity for antibacterial immunotherapy. Then, immune-involved multimodal antibacterial therapy for the treatment of infectious diseases was systematically summarized. Finally, the prospects and challenges of immune-involved combinational therapy are discussed.

**Graphical Abstract:**

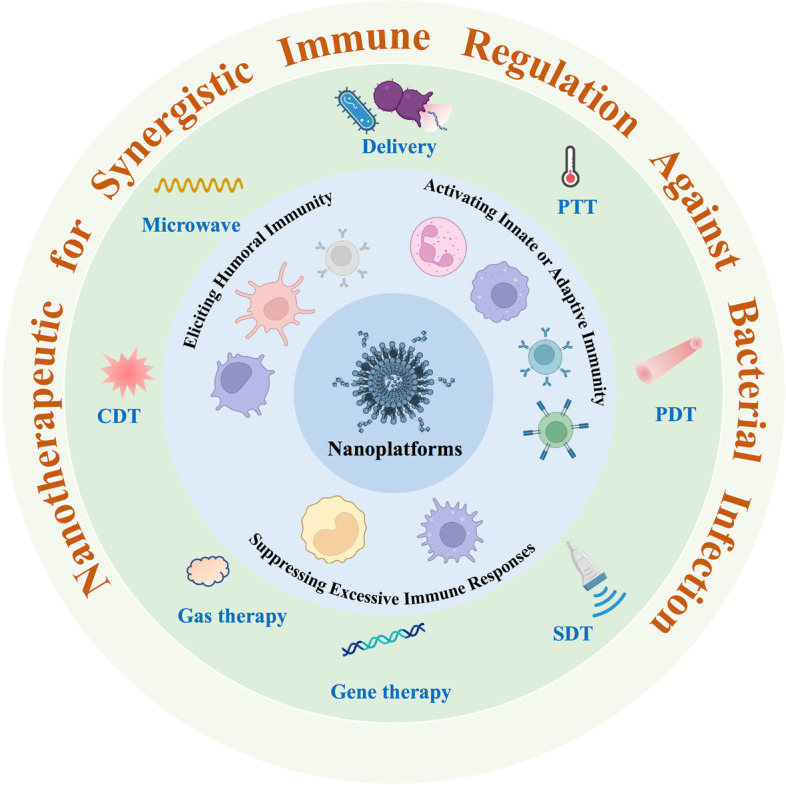

## Introduction

Bacterial infection with high morbidity and mortality has been one of the most severe health issues globally [1, 2]. According to statistics, there were approximately 700,000 deaths in 2015 from antibiotic-resistant bacterial infections, and it is estimated that this death toll will soar to 100,000 by 2050 [3, 4]. Although antibiotics can treat infectious diseases, the overconsumption and abuse of antibiotics will result in the advent of antibiotic-resistant strains and even multiantibiotic-resistant strains, known as ‘superbugs’ [5, 6]. Superbugs can survive even exposure to antibiotics, further making antibiotics less effective [7, 8]. Thus, developing alternative therapeutic strategies or novel combination therapy routes is urgently needed for the treatment of refractory bacterial infections.

In addition to kill bacteria through antibiotics, previous studies have shown that regulating immune responses is crucial in controlling bacterial infections [9, 10]. This indicates that the immune system is important for eliminating the threat of bacteria, and regulating immunity exhibits great practical application value in the treatment of bacterial infection [9]. For example, host-directed therapies have been used for the treatment of sepsis and *Mycobacterium tuberculosis*-associated tuberculosis by targeting the host immune response to infection by augmenting immunity or ameliorating immunopathology [4, 11]. Therefore, revealing the action mechanism of bacteria and the immune system during bacterial infection is beneficial for regulating immunity to remove the invading pathogen and control bacterial infection.

To date, bacterial infections show many hallmarks of immune suppression, indicating that regulating immunity could also be a revolutionary method for treating bacterial infections, including persistent and antibiotic-resistant infections [12–14]. Efficient immune regulation for treating bacterial infection has made great breakthroughs and is being a treatment method with great potential for clinical translation [13, 15]. Multifarious immune regulation methods hold great promise for treating various bacterial infections, including cytokine regulation [16], immune checkpoint blockade (ICB) antibodies [17], antibacterial vaccines [18], macrophage cellular therapy [19], and passive antibody therapy [20]. These have been implemented and have exhibited exciting results in small animal models and in preclinical studies [21, 22]. Among them, antibacterial vaccines generally involving pathogen-associated antigens and immunoadjuvants can stimulate pathogen-specific immune reactions to fight foreign pathogenic bacteria and can potentially achieve long-term immune memory effects to prevent the recurrence of bacterial infection [23]. However, the efficacy and safety of the aforementioned therapeutic methods still need to be improved. For example, the complicated fabrication process, dosage indeterminacy, and limited treatment outcome of antibacterial vaccines lead to an unimpressive antimicrobial immune response. In addition, the enhanced antibacterial immune response of ICB therapy is often accompanied by autoimmune diseases [24, 25]. Therefore, it is urgent to exploit safe and effective avenues to enhance the antibacterial immune response and minimize systemic adverse effects to further enhance the therapeutic effect of infectious disease immunotherapy to alleviate the current crisis of drug-resistant bacteria.

**Fig. 1:**
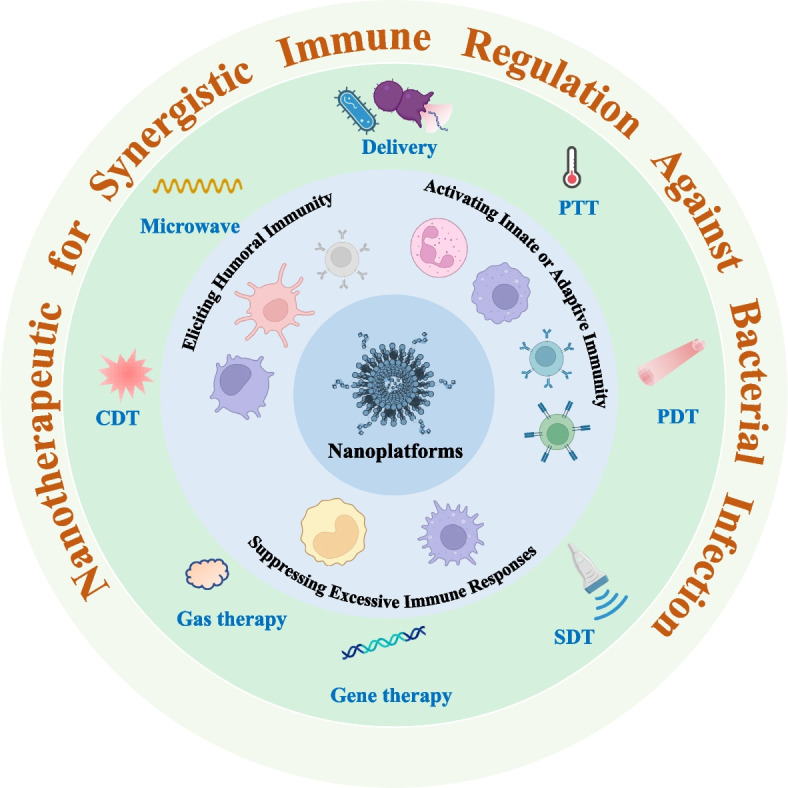
Various nanoplatforms have been applied for enhanced antibacterial immune reactions. These nanoplatforms can deliver immunostimulatory agents to activate antibacterial immunity. Moreover, nanoplatforms can synergistically promote the antibacterial effects and induce antibacterial immune responses in multiple antibacterial therapy strategies, such as photothermal therapy (PTT), photodynamic therapy (PDT), sonodynamic therapy (SDT), gene therapy, gas therapy, chemodynamic therapy (CDT), or microwave therapy

To boost the antibacterial efficacy and ameliorate the adverse effects of infectious disease immunotherapy, various nanoplatforms have been employed for enhanced antibacterial immune reactions (Fig. 1). These nanoplatforms can deliver immunostimulatory agents to activate antibacterial immunity. In addition, nanoplatforms not only synergistically promote the microbial inhibitory effects of multiple antibacterial therapies but also further stimulate the antibacterial immune response [26–28]. Pathogenic bacterial cells that are disrupted by nanoplatform-mediated antibacterial therapies release pathogen-related antigens. These antigens serve as in situ antibacterial vaccines to stimulate a powerful antibacterial immune reaction, further exhibiting huge potential in inhibiting the spread and recurrence of bacterial infection. To implement the aforementioned goal, rationally designed therapeutic systems are desperately needed. Moreover, the spatially and temporally controlled delivery of immunostimulatory agents should be carefully considered to fulfil systemic immune responses while circumventing their own adverse effects. In this review, we summarized the recent advances in nanotherapeutics for synergistic immune regulation against bacterial infection. Then, the predicaments and opportunities of nanotherapeutic-mediated immune regulation will be outlooked. We hope that this review affords novel insights into the field of immuno-antibacterial therapy and its multimodal combined therapy and offers valuable information to promote the development of antibacterial therapy.

## Immune regulation of nanoplatforms

### Mechanisms by which bacteria escape immune system clearance

The local environment of a focal site that includes specific chemical and physical signals is usually referred to as the microenvironment. For example, bacteria in infected tissue can release virulence factors, such as hemolysin and toxins, that form pores, hinder the phagocytic immune response and simulate the immunosuppressive microenvironment [29]. In addition, human infectious diseases are usually divided into chronic and acute infections [30]. Acute infection is easier to treat with antibiotics than chronic infections [31]. Chronic infections are often associated with bacterial biofilm formation. The bacteria located in biofilms are encased in the biofilm matrix, which consists of exopolysaccharides, extracellular DNA, proteins, etc. [32, 33]. The physical barrier formed by biofilms makes it difficult for antibiotics to penetrate [34, 35]. Innate immunity is at the forefront of the body’s fight against invasive pathogens, and can identify and provide defensive protection. Recognizing the receptor (pattern recognition receptor, PRR) that depends on the trigger signal pathway not only reminds the immune system of the existence of infection but also initiates the adaptive immune response by activating antigen-presenting cells [36]. However, biofilm-mediated infections can not only limit antibiotic therapy but also inhibit host innate/adaptive immune responses [37]. Moreover, the biofilm formed by bacteria can inhibit innate immunity through the conversion of macrophages from an inflammatory phenotype to an anti-inflammatory phenotype, thereby inhibiting the phagocytosis and bactericidal activity of macrophages.

In recent years, with the popularity of medical implants, an increasing number of implant infections have been found in clinical treatment [38, 39]. Many pathogens can induce implant-related infections [36, 39]. Implant-related infection generally includes intricate interactions between bacteria, biomaterials and host immune responses [40, 41]. In the absence of foreign bodies, invading bacteria are usually spontaneously eliminated by the host immune defense. In contrast, in implant-related infections, biomaterials trigger local tissue responses, including acute and chronic inflammation, foreign body reactions, granulation tissue formation, and finally fiber encapsulation [42]. This will produce an immunosuppressive niche, that is, a secondary site of resistance, making the implant prone to microbial colonization and infection [43]. Therefore, it is urgent to develop new antibacterial treatment methods in addition to antibiotics.

### Immune regulation pathways

Due to the unique properties of nanomaterials, they have shown great prospects in antibacterial treatment, which provides a new direction for solving bacterial drug resistance. In recent years, nanoplatforms have attracted increasing attention in regulating immunity to bacterial infection [44].

#### Delivery of immune components

These emerging nanoplatforms can deliver immune components to train the immune system and elicit humoral immunity. Current evidence indicates that nanovaccines can maintain the immunogenicity of antigens while reducing adverse effects [45]. In general, nanocarriers preloaded with bacterial subunits and toxoids acting as nanovaccines can activate dendritic cells (DCs), macrophages, B cells and T cells, elicit humoral immunity, and induce a balanced Th1/Th2 response [44, 46].

#### Activation of innate or adaptive immunity

Innate immunity is an effective barrier for multicellular biological recognition and resistance to microbial infection. However, their antibacterial efficacy is limited by insufficient accumulation and inactivation of immune cells. An immunocompromised microenvironment develops when the tissue suffers from a devastating infection. Nanoplatforms can activate innate or adaptive immunity after bacterial infection, promoting the immune system to kill bacteria. Functionalized nanomaterials can promote immune cell (such as macrophages and neutrophils)-secreted chemokines to recruit inflammatory cells into infected tissue to remodel the immunosuppressive environment [47]. Meanwhile, nanoplatforms can promote macrophage M1 polarization to increase macrophage-mediated antibacterial performance [47]. In addition, some nanoplatforms reshaping the infected environment and downregulating the level of myeloid-derived suppressor cells were developed to enhance the therapeutic effect of infectious diseases [28]. Adaptive immunity can achieve long-term protection against bacterial infection. After administration of immunomodulatory nanoagents, patients can harvest immunological memory to prevent recurrence of bacterial infection [48]. Nanoplatform-mediated bacterial immunogenic cell death (ICD) can promote the release of microorganism-associated antigen molecular patterns (MAMPs) and damage-associated molecular patterns (DAMPs). Subsequently, these immunogenic products regulate the polarization of macrophages, promote DC maturation, activate T cells, and induce B-cell-based immune memory [49]. In addition, the abnormal increase in myeloid-derived suppressor cells (MDSCs) also contributed to the immunosuppressive environment at the infection site. Some nanoplatforms can increase M1 polarization of macrophages, reduce MDSC levels, and activate T cells to enhance antibacterial immunotherapy [28].

#### Amelioration of excessive inflammatory reactions

Nanoplatforms can be designed to regulate excessive inflammatory reactions. Patients with chronic infections and sepsis may suffer an overactive inflammatory reaction [50, 51]. For example, in patients with sepsis, excessive inflammatory reactions can trigger cytokine storms, further resulting in cell damage and even organ dysfunction [51]. Due to the adjustability of nanoplatform properties, they can also regulate the overactivation of the immune system and excessive inflammatory reactions. For example, nanoplatforms can reduce the level of M1 macrophages, induce the polarization transformation of M1 to M2 macrophage cells and suppress cytokine storms, which can ameliorate infectious inflammatory disease and even improve wound healing [52]. In addition, nanoplatforms can enhance the levels of regulatory T cells (Tregs) or prevent elevated leukocyte and neutrophil levels to regulate excessive inflammatory reactions [53, 54].

## Delivery of nanovaccines for regulating antibacterial immunity

Previous results confirm that attenuated or inactivated bacteria, partial pathogen components (*e.g.*, bacterial outer membrane, polysaccharides, proteins, peptides or nucleic acids), and toxoids can serve as antigens to stimulate innate and acquired immune responses for the elimination of pathogens in the body [55]. These components can be loaded into nanocarriers as nanovaccines to regulate antibacterial immunity. The advantages of nanovaccines mainly include the following: (I) nanocarriers can avoid or alleviate the rapid inactivation of antigens caused by degradation and increase vaccine stability; (II) nanocarriers offer a satisfactory adjuvant capability and facilitate the activation of antigen presenting cells (APCs); and (III) nanometer-scale of nanocarriers enhance the enrichment of antigens in lymph nodes, further boosting the immune reaction.

### Bacterial membrane-modified nanovaccines

**Fig. 2:**
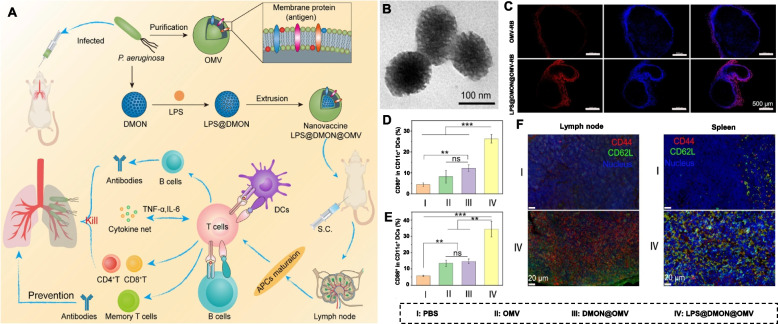
Immuno-antimicrobial regulation of bacterial membrane-coated nanovaccine for the treatment of bacteria. **A** Illustration diagram of a nanovaccine encapsulated by bacterial outer membrane vesicles (OMVs) for the treatment of *Pseudomonas aeruginosa* pneumonia. **B** TEM of LPS@DMON@OMV. **C** CLSM of inguinal lymph nodes (LNs), which were isolated at 12 h after LPS@DMON@OMV and free OMV-labeled RB injection. **D**, **E** Flow cytometry results of surface maturation markers (CD80 and CD86) on CD11c^+^ DCs. **F** Immunofluorescence staining of central memory T cells in inguinal LNs and spleen [56]. Copyright©2022 Elsevier B.V

Biomembrane engineering is employed in vaccine research due to homologous cell membranes containing specific antigens, which have strong immunogenicity. Motived by this phenomenon, the combination of bacterial outer membrane vesicles (OMVs) and nanomaterials to prepare nanovaccines can be used to stimulate antibacterial immunity. For example, Han et al. reported an LPS@DMON@OMV nanovaccine for the treatment of *Pseudomonas aeruginosa* pneumonia (Fig. 2A) [56]. In the preparation process, the immunoadjuvant lipopolysaccharide (LPS) was preloaded into dendritic mesoporous organosilica (DMONs). The microbial membrane antigen (OMV) originating from *Pseudomonas aeruginosa* was coated onto the surface of DMONs (Fig. 2B). When administered to mice, LPS@DMON@OMV targeted and activated LNs (Fig. 2C), and stimulated the activation and maturation of DCs (Fig. 2D, E). Moreover, the antibody titer in the LPS@DMON@OMV-treated group was much higher (180-fold) than that in the OMV-treated group. Meanwhile, more central memory T cells were present in the mice treated with LPS@DMON@OMV (Fig. 2F). The combination of T cells and antibodies could cure pneumonia. In addition, immune memory was harvested, which can prevent the occurrence of bacterial infection. This works indicates that nanovaccines derived from OMVs possess tremendous potential for bacterial infection control.

### Immune cell membrane-modified nanovaccines

**Fig. 3:**
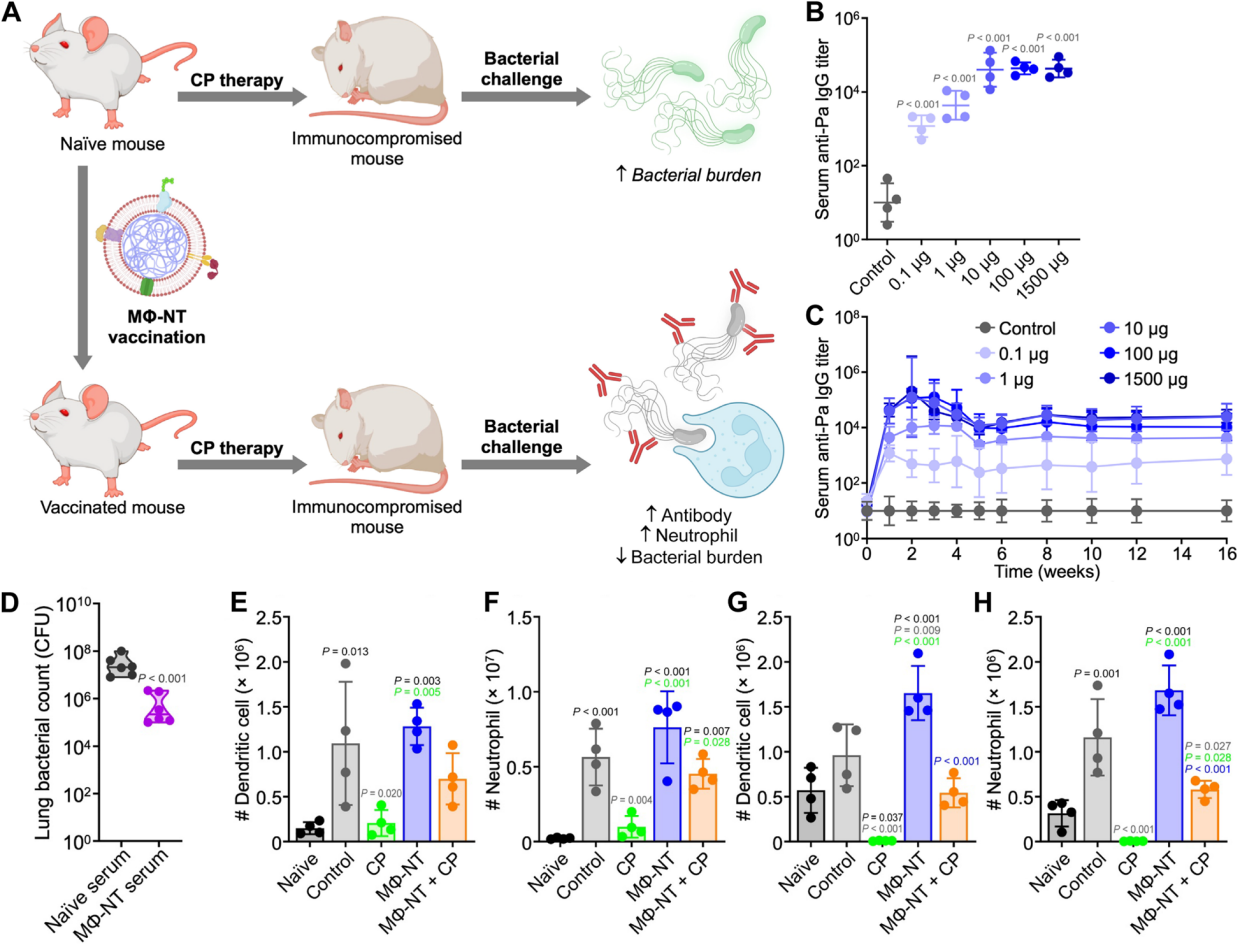
Mice vaccinated with the MΦ-NT nanovaccine can obtain rapid and long-lasting immunity. **A** Illustration diagram of an immunocompromised mouse vaccinated with MΦ-NT that can obviously combat bacterial infections. **B** Serum anti-*P. aeruginosa* IgG at day 7 and after vaccination with different doses of MΦ-NT and **C** over 16 days after treatment with different concentrations of MΦ-NT on days 0, 7, and 14. **D** Bacterial load in mice intratracheally challenged with 10^7^ or 10^6^ CFU *P. aeruginosa*. **E** DCs and **F** neutrophils per lung 1 day after the mice were infected with 10^7^ CFU *P. aeruginosa*. **G** DCs and **H** neutrophils per 1 mL of blood 1 day after the mice were infected with 10^6^ CFU *P. aeruginosa* [10]. Copyright © 2022 Zhou et al*.*, American Association for the Advancement of Science

**Fig. 4:**
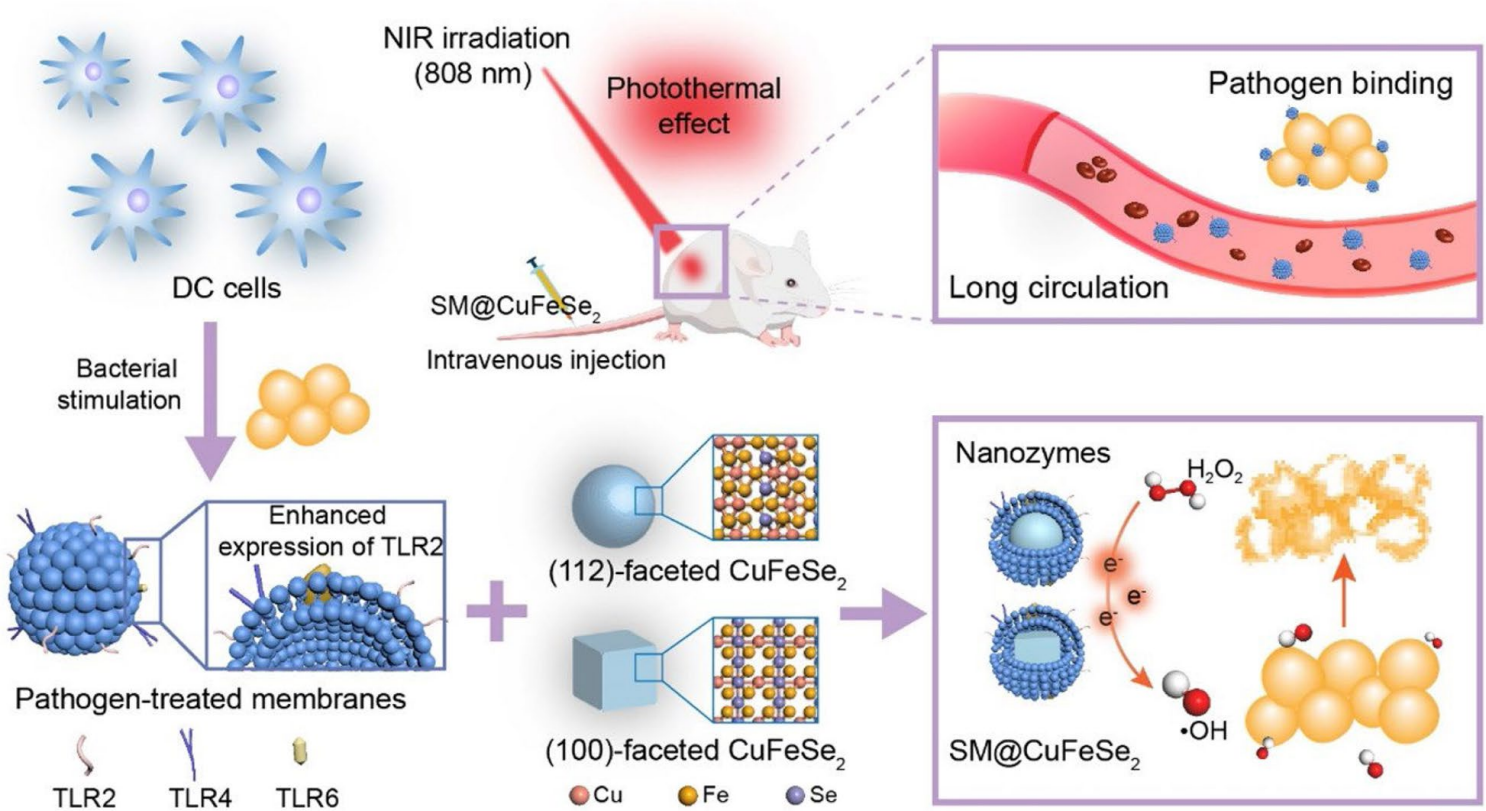
Dendritic cell membrane-modified nanovaccines. DC membrane-functionalized CuFeSe_2_ with photothermal antibacterial properties enhances immune evasion and antimicrobial properties [59]. Copyright©2021, American Chemical Society

Motivated by recent achievements in nanotechnology, the microbial membrane can be extracted and wrapped on the surface of nanomaterials to act as an antimicrobial vaccine. Similar to OMVs, cell membrane-encapsulated nanoparticles integrated with pathogen-associated antigens could also act as vaccines for preventing infectious diseases by inducing the body’s immune system for targeted elimination of pathogen-associated antigens and even intact bacteria [57]. Currently, scientists have found that immobilizing bacterial toxins to immune cell membrane carriers can boost the immune system’s ability to eliminate drug-resistant bacteria [10]. These nanotoxoid vaccines offer a new route to alleviate the threat caused by drug-resistant bacteria. For instance, Zhang’s team developed a biomimetic toxoid nanovaccine (MΦ-NT) to address the problem that immunocompromised patients are susceptible to bacterial infections (Fig. 3A) [10]. In the synthesis process, the macrophage membrane was extracted and wrapped around the surface of carboxyl-terminated poly(lactic-co-glycolic acid) (PLGA) NPs. By taking advantage of the properties of the macrophage membrane, the toxin (Pas) secreted by the *Pseudomonas aeruginosa* P4 clinical isolate could immobilize stealthily and safely present to immune cells because the macrophage membrane has toxin neutralization ability [45, 58]. After giving MΦ-NT to immunodeficient mice, they developed rapid immunity and long-term resistance to fatal infections (Fig. 3B-D). MΦ-NT presented satisfactory therapeutic effects in the treatment of pneumonia and septicaemia by increasing DC and neutrophil counts and antibody titres (Fig. 3E-H). These results confirmed that the nanotoxoid vaccines have tremendous clinical translational prospects, especially in immunodeficient patients. Hou et al. prepared 100-faceted CuFeSe_2_ nanocubes and 112-faceted CuFeSe_2_ nanosheets (Fig. 4) [59]. The Fenton-like catalytic activity of the CuFeSe_2_ nanocube was higher than that of the CuFeSe_2_ nanosheet due to the (100) facets possessing a low energy barrier, which benefited the occurrence of the Fenton-like reaction. To endow CuFeSe_2_ with bacterial affinity and immune evasion performance, a pathogen-activated dendritic cell membrane was extracted and wrapped onto the surface of CuFeSe_2_ nanocubes (SM@CuFeSe_2_). After intravenous administration of SM@CuFeSe_2_, SM@CuFeSe_2_ can target and accumulate in infected tissue. In addition, after exposure to an 808 nm laser, almost all *Staphylococcus aureus* was inactivated. Due to the bacterial targeting of the Toll-like receptor 2 (TLR2) receptor in the DC membrane, SM@CuFeSe_2_ nanocubes could achieve specific photothermal-enhanced chemodynamic therapy of bacteria-infected osteomyelitis, further indicating the antibacterial application potential of SM@CuFeSe_2_ nanocubes.

### Synthetic pathogen-associated molecular patterns (PAMPs)-loaded nanovaccines

**Fig. 5:**
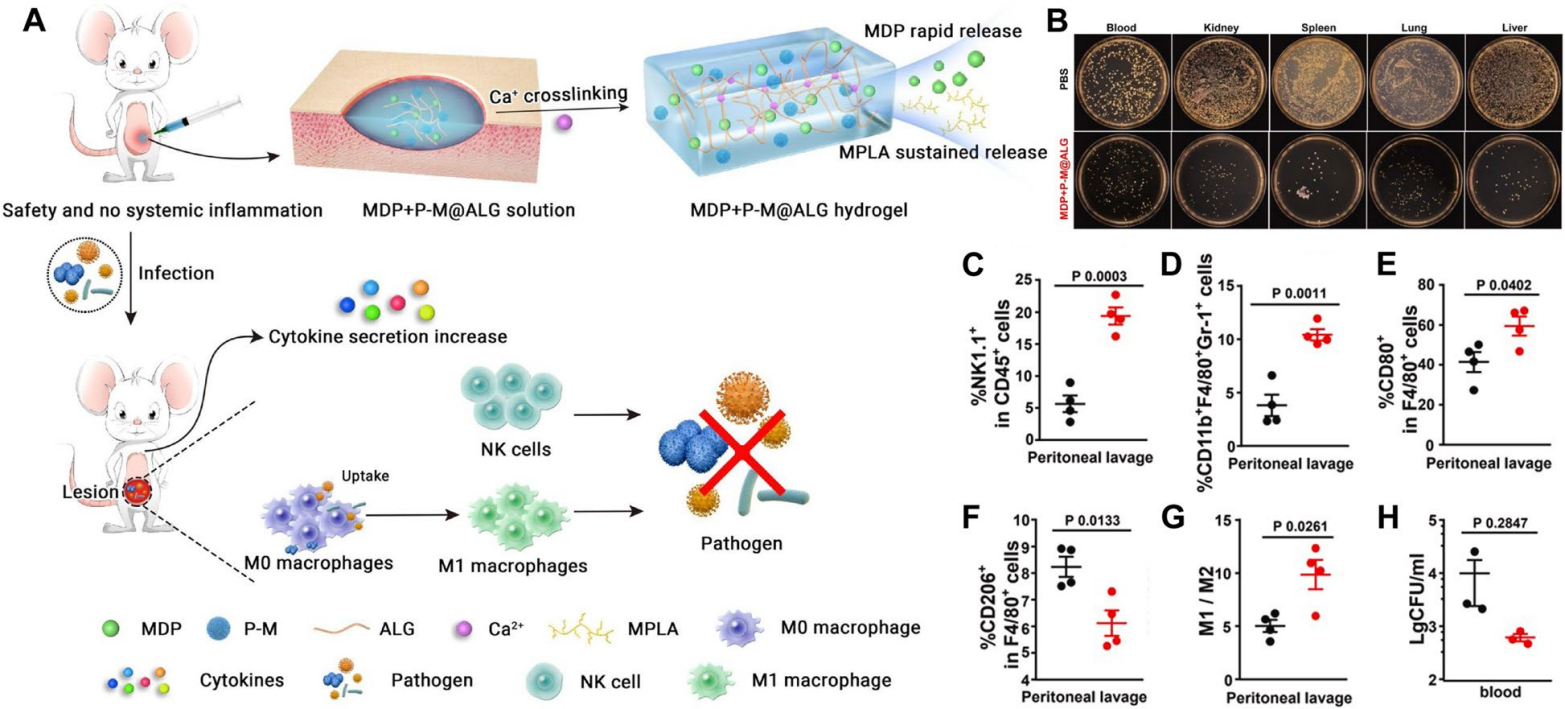
Two-phase releasing MDP + P-M@ALG hydrogel for the immune-antimicrobial therapy of sepsis. **A** In vivo protection against repeated bacterial infection in surviving mice. **B** Representative images of *E. coli* colonization in the major organs of the mice after *E. coli* infection. The percentages of **C** NK cells, monocytes, **D** CD11b^+^F4/80^+^Gr-1^+^ cells, **E** M1 macrophages, **F** M2 macrophages and **G** M1:M2 ratios in peritoneal lavage of the mice at 8 h after the second *E. coli* challenge. **H** Statistical data of *E. coli* colonization in the blood of the mice [60]. Copyright©2021 Elsevier Ltd

The activation of PRRs can trigger cytokine release to facilitate the function of immune cells, reinforce anti-infective activity in the body and remove invading pathogens. Representative synthetic PAMPs that activate PRRs in the clinic include imiquimod and monophosphoryl lipid A (MPLA), which are Toll-like receptor (TLR) agonists, while muramyl dipeptide (MDP), a nucleotide-binding oligomerization domain (NOD)-like receptor agonist, participates in protection against infectious diseases [60]. Based on the above results, Liu et al. developed a “two-phase releasing” MDP + P-M@ALG hydrogel for sepsis therapy (Fig. 5A, B) [60]. MPLA was loaded into PLGA NPs (P-M) to realize the sustained release of MPLA. Under the initiation of Ca^2+^, the P-M NPs, alginate (ALG) solution containing muramyl dipeptide (MDP) and P-M NP immunomodulatory agents could form MDP + P-M@ALG hydrogels. After subcutaneous injection of the MDP + P-M@ALG hydrogel, MDP could be released rapidly to boost phagocytosis and the bactericidal performance of macrophages, and consequently, immediate protection was aroused. Subsequently, the slow release of MPLA from MDP + P-M@ALG could provide long-range protection against sepsis by increasing the generation of proinflammatory cytokines, accelerating macrophage differentiation, and elevating the content of natural killer (NK) cells in the infected site (Fig. 5C-H). After hydrogel administration, the mice with sepsis had significantly higher survival rates, and the surviving mice could resist secondary infection. In addition to Liu’s research group, the efficacy of polysaccharide and lipopeptide vaccines was also confirmed by Toth, Alugupalli, Stephenson, Leadbetter et al*.* [46, 61, 62]*.* These works indicate that peptides and lipid antigens can be used as reliable vaccines for bacterial infection management.

### Tumor exosome delivery nanoplatforms for treating sepsis

**Fig. 6:**
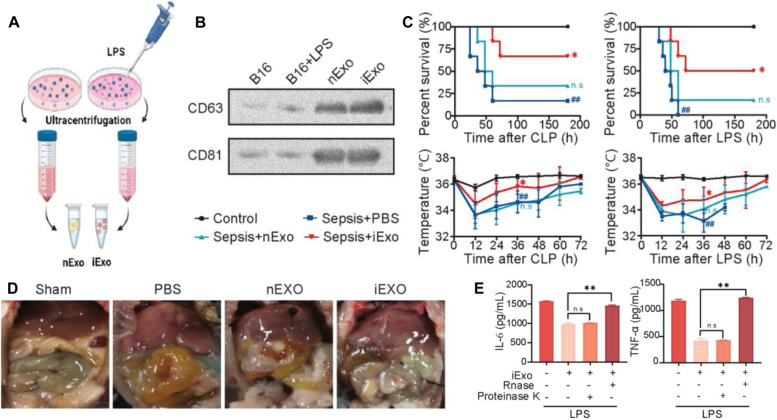
Tumor exosome delivery nanoplatforms to suppress sepsis. **A** Normal (nExo) and LPS-induced (iExo) exosome purification processes. **B** Exosome marker analysis. **C** Survival rate and temperature profiles after mice with CLP-induced (left) or LPS-induced sepsis (right) were treated with PBS, nExos, and iExos. **D** Representative ventral images after the mice with CLP-induced sepsis received different treatments. **E** The effect of iExos on the levels of inflammatory factors in macrophages treated with LPS [52]. Copyright©2022 Li et al*.*, Advanced Materials published by Wiley–VCH GmbH

Sepsis is a systemic infectious disease that results from the spread of pathogens and their toxins into the bloodstream. This process is often accompanied by overactive immune reactions and even organ dysfunction and shock*,* which cause serious harm to the patient. Therefore, suppressing immune overactivation is a potential route for the treatment of sepsis. In general, people with cancer show an immunosuppressive state, which indicates that this immunosuppression can prevent immune overactivation related to sepsis. Thus, patients with sepsis have a higher survival rate in the cancer patient population [52, 63]. Inspired by the above observed research phenomenon, Sun et al. developed a tumor exosome delivery nanovaccine for the management of sepsis (Fig. 6A-D) [52]. In the sepsis model, the exosomes (iExos) produced from tumor cells pretreated with lipopolysaccharide (LPS) presented a protective outcome because the miRNAs located in the iExos could downregulate inflammatory cytokines (*e.g.,* IL-6 and TNF-α) (Fig. 6E). By mimicking iExos using hyaluronic acid-polyethylenimine nanocarriers, nanocarriers loaded with specific miRNAs could relieve sepsis in mice and monkeys, indicating that RNA vaccines offer an alternative therapeutic avenue for sepsis therapy.

### Nanocarriers for antibacterial drug delivery and synergistic immune regulation

**Fig. 7:**
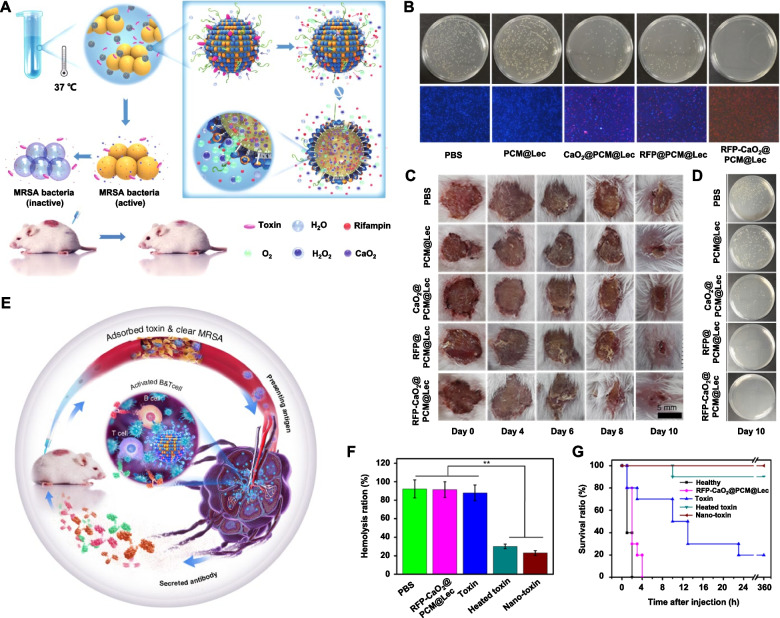
Stimulus-responsive antibiotic delivery nanoreactors. **A** RFP-CaO_2_@PCM@Lec nanoreactors for the treatment of bacterial infection. **B** Plate images and live/dead analysis (red fluorescence, dead cell). **C** Photographs of wounds infected by MRSA and **D** grown colonies after different treatments. **E** RFP-CaO_2_@PCM@Lec nanoreactors trigger immune reactions to boost the therapeutic outcome. **F** Degrees of hemolysis ratios in different groups. **G** Survival rates of mice following an *i.v.* injection of α-toxin [67]. Copyright © 2019, The Wu et al., Springer Nature

Although antibiotics have made remarkable progress in the treatment of infectious diseases, the occurrence of superbugs attenuates the effect of antibiotic treatment [5]. In addition, the toxin produced by pathogens can revamp the immune signaling pathway, further inhibiting phagocyte bactericidal performance by reducing the generation of reactive oxygen species (ROS) and nitric oxide (NO) antimicrobial substances and suppressing the phagocytic function of immune cells [64, 65]. Thus, neutralizing bacterial toxins can rejuvenate host immune reactions for the treatment of infectious diseases [66]. For instance, Han et al*.* designed and synthesized a pathogen toxin-induced rifampicin (RFP) release nanoplatform (RFP-CaO_2_@PCM@Lec) (Fig. 7A, B) [67]. RFP-CaO_2_@PCM@Lec was fabricated after RFP antibiotic and calcium peroxide (CaO_2_) were coated with low melting point phase change materials (PCM) and liposomes. At the site of infection, the toxin secreted by MRSA was trapped in RFP-CaO_2_@PCM@Lec. After the toxin intercalated into liposomes, a pore was created to allow water to seep into the nanoparticle and react with CaO_2_, further producing hydrogen peroxide (H_2_O_2_). The oxygen originating from the decomposition of H_2_O_2_ could drive RFP release for the treatment of infected wounds (Fig. 7C, D). In addition, RFP-CaO_2_@PCM@Lec with strong toxin-neutralizing performance blocked toxin-induced hemolysis (Fig. 7E, F). The toxin and RFP-CaO_2_@PCM@Lec compound enhanced the host immune response to the toxin (Fig. 7G). Thus, the targeted therapeutic agent RFP-CaO_2_@PCM@Lec presents superior therapeutic performance by stimulus-responsive delivery of antibiotics and immunoregulation.

In addition, it needs to be considered that some antibiotics can affect the body’s immune response via different mechanisms during treatment. For example, ciprofloxacin treatment can directly reduce the phagocytic activity of immune cells by inhibiting mitochondrial respiration [68]. Therefore, some polymer antibacterial agents can be delivered via nanoplatforms to protect against microbial infections. Dai et al*.* used N,N′-methylene bisacrylamide (MBA), 1-(2-aminoethyl)piperazine (AEPZ), and agmatin (Agm) as raw materials to synthesize a hydrophilic branched polymer (poly(MBA-AEPZ)-Agm) by Michael addition polymerization for chemoimmunotherapy against microbial infections. The Agm was introduced into the cationic antibacterial poly(amino ester) to activate macrophages. After incubation with poly(MBA-AEPZ)-Agm1, high levels of inflammatory factors (NO, IL-1β, and TNF-α) were present in macrophages, and the bacteria could be selectively suppressed with the aid of macrophages. Using this chemotherapy/immunotherapy dual-modal therapeutic agent, subcutaneous *S. aureus* infection can be successfully treated [69].

### Nanoplatform-mediated gene therapy regulating antibacterial immunity

Over the past decades, gene therapy has achieved great progress in infectious diseases and has emerged as an alternative treatment option for infectious diseases. The introduction of target genes either blocks or inhibits the reproduction of pathogenic bacteria by intervening with gene transcription and translation or the function of posttranslational proteins or prevents the spread and diffusion of pathogenic bacteria at the extracellular level by continuously secreting inhibitory proteins or triggering specific immune reactions in vivo [70]. However, the therapeutic effect of gene therapy is limited due to its low gene transfer efficiency. Thus, the application of delivery nanoplatforms can significantly enhance the therapeutic effect of infectious diseases. In 2021, Sailor’s group fabricated fusogenic porous silicon nanoparticles with activated macrophage-targeting performance for shielding the proinflammatory cytokine signal by delivering siRNA targeting the *Irf5* gene [71]. By introducing siRNA into activated macrophages, the side effects caused by excessive inflammatory reactions were minimized, and the body’s immune system was significantly enhanced to effectively eliminate pathogens [72]. Subsequent in vivo studies have shown that the combination of siRNA interference technology and immunotherapy exhibited a significant therapeutic effect both in *Pseudomonas aeruginosa* (PA01) lung infection and methicillin-resistant *Staphylococcus aureus* muscle infection mouse models, indicating that this treatment strategy had broad spectrum antibacterial performance and could break through bacterial resistance in vivo.

As refractory implant infection intensifies, it is difficult to treat due to the inefficient delivery of antibiotics to infected tissues, recurrence of surviving pathogens, immune escape and regional immunosuppression. Gene therapy can not only modulate the function of immune cells but also express antibacterial drugs, such as antimicrobial peptides, in the recipient cell. For example, Yue et al*.* constructed an ROS-responsive TSPBA-PVA hydrogel for antimicrobial peptide (LL37) and plasmid delivery [28]. The surface of the ZIF-8 porous nanomaterial preloaded with the LL37 plasmid was coated with polydopamine for late surface modification of the antimicrobial peptide LL37. The antibacterial peptides on the surface of nanomaterials could effectively eliminate *Staphylococcus aureus* in both intracellular and extracellular environments. Furthermore, LL37 continued to be expressed after the LL37 plasmid was successfully transfected, which effectively avoided the recurrence of infection. Moreover, the resulting LL37@ZIF8-LL37 NPs could alleviate infection-induced immunosuppression by deterring the abnormal increase in myeloid-derived suppressor cells. Taken together, the integration of gene therapy and immunotherapy can be used in the management of implant-related infectious diseases.

## Exogenous-responsive nanoplatforms

### Phototherapeutic antibacterial nanoplatforms for synergistic immune regulation

Recent works have indicated that phototherapy, such as photothermal therapy (PTT) or photodynamic therapy (PDT), can reinforce the ICD reaction against persistent infection [73]. Hyperthermia and oxidative stress induced by nanoplatform-mediated PTT and PDT, respectively, can act as exogenous stress to activate the immune system by triggering immunogenicity [74–76]. Many emerging antibacterial nanoplatforms with immunotherapeutic effects can also stimulate antigen-presenting cells (such as macrophages or DCs) to produce adjuvant immunotherapy that can trigger bactericidal behavior to solve the challenge of recurrent infections.

#### Photothermal antibacterial nanoplatforms

It is widely known that macrophages can differentiate into two types of cells (M1- and M2-type macrophages) in different environments. M1-type macrophages are beneficial for clearing pathogens, while M2-type macrophages are beneficial for repairing wounds [77]. During PTT, the hyperthermia induced by photothermal agents under laser conditions can destroy bacteria and produce debris to trigger M1 macrophage polarization. For example, Chang and colleagues synthesized polydopamine and glycol chitosan dual functionalized copper-doped mesoporous silica (MCS@PDA@GCS). When MCS@PDA@GCS was applied to an infected wound, the protonated MCS@PDA@GCS with a positive charge in the acidic infected wound was enriched on the surface of pathogens. The photothermal antibacterial activity of MCS@PDA@GCS was activated upon exposure to an 808 nm laser. Through the combination of photothermal and Cu ion antibacterial properties, MCS@PDA@GCS exhibited durable and efficient antibacterial properties toward planktonic and biofilm bacteria. Moreover, MCS@PDA@GCS enhanced immune-mediated antibacterial activity by inducing the formation of M1-type macrophages. In addition, the ions released from MCS@PDA@GCS could promote endothelial cell migration and vascular regeneration. Then, multifunctional MCS@PDA@GCS could effectively kill bacteria and promote infectious wound healing in vivo.

**Fig. 8:**
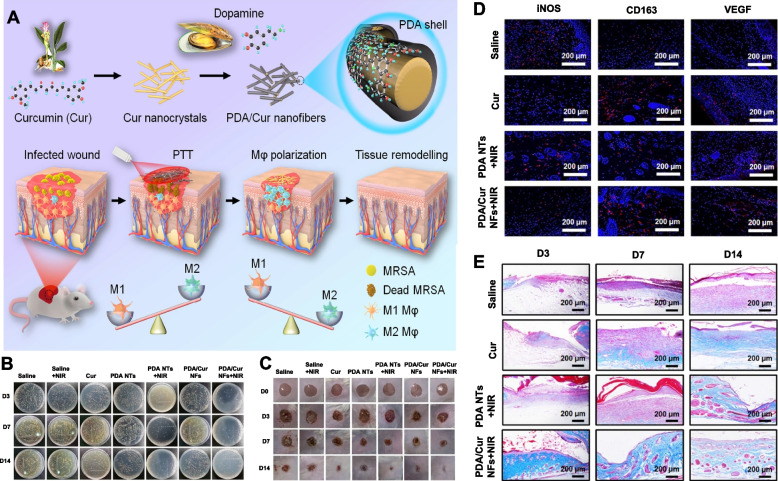
PDA/Cur nanofibers for photothermal antibacterial therapy and regulation of macrophage polarization in diabetic infected wounds. **A** Schematic illustration of photothermal antibacterial and M2 polarization for treating MRSA-infected diabetic wounds. **B** Photographs of the bacterial colony and **C** representative images of the wound healing process of mice with different treatments. **D** Representative immunofluorescence images showing iNOS, CD163, and VEGF, **E** Masson staining of wounds in different treatment groups [78]. Copyright©2021, The Xu et al*.,* Springer Nature

In general, when M1-type macrophages remove invading pathogenic bacteria and damaged tissues, the macrophages are polarized toward M2-type macrophages, which possess anti-inflammatory and pro-tissue regenerative activity, further avoiding excessive inflammation and expediting vascular regeneration and cell propagation during the proliferative phase of wounds. Unfortunately, in patients with diabetes, M1 macrophages fail to convert to M2 macrophages, resulting in the development of chronic inflammatory wounds, making the wound hard to heal. Therefore, to promote chronic wound healing, it is necessary to develop therapeutic agents that can induce macrophages toward the M2 phenotype at the proper time. For example. Chen et al. developed polydopamine-coated curcumin (Cur) nanofibers (PDA/Cur NFs) for photothermal antibacterial therapy and macrophage programming (Fig. 8A) [78]. The PDA shell with excellent photothermal performance could kill the pathogen under laser exposure (Fig. 8B, C). Thereafter, Cur released from PDA/Cur NFs promoted cell reproduction and facilitated M2-type macrophage polarization. In diabetic infected wounds, PDA/Cur NFs could accelerate wound healing by the combination of photothermal antibacterial therapy and immunoregulation due to photothermal sterilization and nascent M2-type macrophages with reinforced collagen deposition, vasculogenesis, and cell multiplication (Fig. 8D, E). In another work, Fan and colleagues further fabricated an anti-inflammatory hydrogel (HTHE-M@D) for diabetic wound therapy by using tyramine-modified collagen (HLC-TA), epigallocatechin gallate-modified hyaluronic acid (HA-EGCG), and mesoporous PDA nanoparticles preloaded with deferoxamine (M@D) [79]. They found that the pathogens were inhibited, and more blood vessels and M2-type macrophages were present after the administration of HTHE-M@D, which further boosted diabetic wound healing through combination treatment with photothermal antibacterial therapy and immunomodulation.

Apart from diabetes infection, periodontitis is a common chronic disease in adults [80]. Usually, periodontitis is accompanied by an excessive inflammatory response, which is exacerbated by high levels of ROS. Recently, Li research team used mesoporous Prussian blue (MPB) nanoparticles to deliver the immunomodulatory agent baicalein (BA) [81]. Due to the antioxidant and anti-inflammatory activity of BA, the presence of MPB-BA was beneficial to the polarization of M1-type to M2-type macrophages by eliminating intracellular ROS. Molecular biology studies indicated that MPB-BA reduces inflammation via the Nrf2/NF-κB signaling pathway. Moreover, by combining the antibacterial activity of BA and the photothermal antibacterial activity of PB, MPB-BA presented an exceptional therapeutic effect in periodontitis. This evidence demonstrates that photothermal-assisted immunotherapy holds great potential in the treatment of infectious diseases.

#### Photodynamic antibacterial nanoplatform

Antibacterial photodynamic therapy (aPDT) was implemented over 100 years ago and has been used to treat clinical diseases, such as leishmaniasis, acene, infected leg ulcer, and brain *abscess* [82, 83]. Upon excitation with an appropriate light source, photosensitizers can produce substantial ROS to kill the bacteria [84–86]. Apart from destroying cell membrane integrity, aPDT can also induce an in vivo immune response. Studies have indicated that the permeability of neutrophils in the area of infectious arthritis was enhanced after treatment with aPDT. Neutrophils can not only secrete hydrolytic enzymes and antimicrobial peptides to kill the pathogen but also release prostaglandins, cytokines, leukotrienes, etc*.,* to promote inflammation [87]. The accumulation of neutrophils at the infected site is a requisite for aPDT-mediated removal of bacterial infections [88]. In addition to neutrophils, macrophages can also be activated and recruited by aPDT to enhance immunity. Macrophages are antigen-presenting cells that ingest antigens and submit them to T lymphocytes in the development of an adaptive immune response. Moreover, the chemokines and cytokines secreted by macrophages are also involved in aPDT-mediated inflammation. Therefore, the host immune system can be stimulated to combat bacterial infection. Many research teams have confirmed that aPDT-mediated immunotherapy can significantly enhance the antibacterial therapeutic effect.

**Fig. 9:**
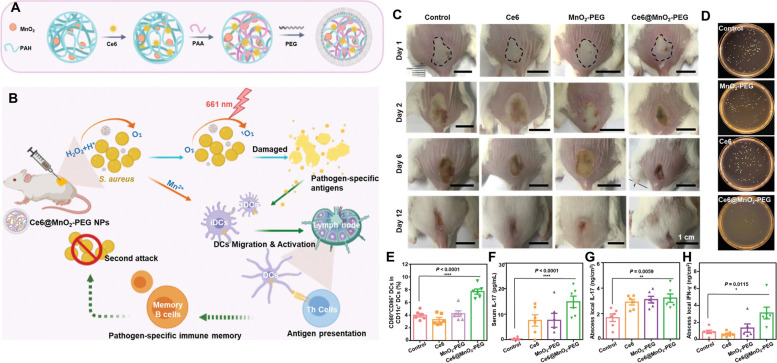
Photodynamic antibacterial nanoplatform. **A** Schematic illustration of the synthesis of Ce6@MnO_2_-PEG NPs. **B** Schematic illustration of Ce6@MnO_2_-PEG NPs boosting aPDT of *S. aureus* infections by relieving abscess hypoxia and reinforcing protective immune responses. **C** Representative photographs of *S. aureus*-infected mice after different treatments. **D** Photographs representing *S. aureus* colonies in the four groups on Day 5. **E** DC maturation induced by Ce6@MnO_2_-PEG-based PDT. **F** Serum IL-17, **G** local IL-17 and **H** IFN-γ levels in the abscess lesions on Day 5 [48]. Copyright©2020 WILEY–VCH Verlag GmbH & Co. KGaA, Weinheim

After aPDT, the release of bacterial antigens can serve as a prophylactic vaccine to induce an immune response, which gradually became a popular area. Immunoadjuvants are generally used to potentiate the host immune response toward antigens. Liu et al. utilized a chlorine e6 (Ce6) photosensitizer and polyethylene glycol (PEG)-functionalized manganese dioxide nanoparticles (MnO_2_ NPs) to prepare Ce6@MnO_2_‐PEG NPs (Fig. 9A, B) [48]. Hydrogen peroxide (H_2_O_2_) from the wound site could be degraded by Ce6@MnO_2_‐PEG NPs, alleviate the hypoxic microenvironment of *Staphylococcus aureus* abscesses, and reinforce the antibacterial activity of aPDT in vivo (Fig. 9C, D). Furthermore, the combination of bacterial antigens generated from aPDT and the immunoadjuvant‐like property of manganese ions could trigger the host immune response and endow the immune system with immunological memory to avoid the recurrence of the same pathogen infection (Fig. 9E). However, superabundant ROS can not only kill the pathogen but also lead to surrounding normal tissue damage during aPDT, which seriously affects the application of aPDT in periodontal diseases. Moreover, a high level of ROS can activate macrophages, which causes the lesion site to be in an inflammatory state and is not beneficial to tissue regeneration at the injured site. To solve this contradiction, Sun et al*.* constructed a CeO_2_@Ce6 nanocomposite for ROS-mediated disinfection and inflammation regulation [89]. Upon irradiation with a 630 nm laser, aPDT was executed for the eradication of pathogenic bacteria, and then the extra ROS were scavenged by nanoceria to regulate the host immune response by inducing M2-type macrophage polarization and reducing M1-type macrophage activation. Additionally, the ROS level in the infected tissue could be modulated permanently due to the reversibility of the Ce valence state in CeO_2_@Ce6. For these reasons, the damaged tissue could quickly undergo remodeling and repair. These results indicated that CeO_2_@Ce6 with photodynamic antibacterial and anti-inflammatory effects has good clinical application prospects in chronic wound infection.

In addition to CeO_2_ with anti-inflammatory properties, some natural organic small molecules derived from traditional Chinese medicine also have anti-inflammatory properties. Therefore, nanotherapeutics with photothermal antibacterial and anti-inflammatory activities can be developed after natural organic small molecules are loaded onto photothermal nanocarriers. For example, Liu et al. developed an MSN-PEG@AS/BP-based spray for accelerating wound healing. In their work, astragaloside IV (AS)-functionalized mesoporous silica nanoparticles (MSNs) were loaded onto black phosphorus (BP) nanosheets [90]. The photothermal activity of black phosphorus (BP) can be triggered for the removal of 99% of pathogenic bacteria and simultaneously promote the release of AS to remodel the wound microenvironment. Due to the proangiogenic and anti-inflammatory activities of AS, fibroblast propagation and wound angiogenesis were enhanced, and the transformation of macrophages at the wound site into the M2 type was enhanced to facilitate extracellular matrix deposition and neovascularization of the wound. This evidence indicated that MSN-PEG@AS/BP-based spray may be a potential therapeutic for chronic infected wounds by the combination of photothermal antibacterial therapy, pharmacotherapy and immunomodulation therapy.

#### Photothermal/photodynamic antibacterial nanoplatform

Photothermal and photodynamic therapy have been used for bacterial biofilm removal [91]. However, unimodal treatment requires a high temperature (over 70 °C) and abundant ROS to eliminate the mature biofilm, which will cause damage to adjacent normal tissue [7]. Thus, multimodal combination therapy is more prevalent and achieves significant success because the combination strategy can heighten antibacterial performance and minimize adverse effects by lowering the therapeutic dosage and laser power [92]. However, some challenges still need to be settled, especially the existence of a biofilm barrier hindering the penetrability of therapeutic agents, heat and ROS. In addition, to expedite the healing of infected wounds, the immune microenvironment at the site of infection needs to be appropriately regulated according to different infectious diseases [50]. For example, excessive immune reactions are observed in periodontal infections, while immunosuppression is present in implant-related bone infections. Therefore, using nitric oxide to regulate M1/M2 macrophage polarization is extremely attractive [93]. The formation of polymicrobial biofilms on teeth is the main cause of periodontitis. To effectively treat periodontal disease, therapeutic agents preferably have antibiofilms and relieve excessive inflammation. To achieve this goal, Wang et al. employed mesoporous silica-functionalized gold nanorods to load ICG and SNO to construct an NIR-triggered multifunctional antibiofilm nanotherapeutic platform [94]. Upon exposure to an 808 nm laser, photothermal and photodynamic therapies were activated for the obliteration of biofilms. Moreover, the NO donor (SNO) could be triggered by hyperthermia to release NO gas to relieve the inordinate immune reaction by destroying the assembly of the NLRP3 inflammasome and suppressing the NF-κB pathway. However, in the biofilm-based implant infections, an immunosuppressive phenomenon is observed due to biofilm environment is conducive to the transformation of macrophages into M2-type macrophages and then inhibits the removal of bacteria by the body’s immune system. To reverse the immunosuppressive environment and overcome the biofilm barrier, Wu et al. developed a multifunctional coating for the treatment of implant-related bone infections [95]. In their work, red phosphorus, PCP hydrogel (poly(vinyl alcohol, chitosan, and polydopamine-based hydrogel), and RSNO were successively functionalized on the surface of a titanium implant (Ti-RP/PCP/RSNO). The photothermal and photodynamic performance of red phosphorus was initiated under 808 nm laser exposure. Moreover, abundant NO was generated and reacted with superoxide (^•^O_2_^–^) to produce peroxynitrite (^•^ONOO^–^) for methicillin-resistant *Staphylococcus aureus* biofilm eradication. Furthermore, in the absence of laser treatment, minor amounts of NO gas originating from RP/PCP/RSNO could boost bone regeneration and M1-type macrophage differentiation by increasing the transcription level of the *Ocn* and *Opn* genes and the translation level of TNF-α. The ex vivo and in vivo evaluations indicated that Ti-RP/PCP/RSNO could be employed for the management of implant-related bone infections.

**Fig. 10:**
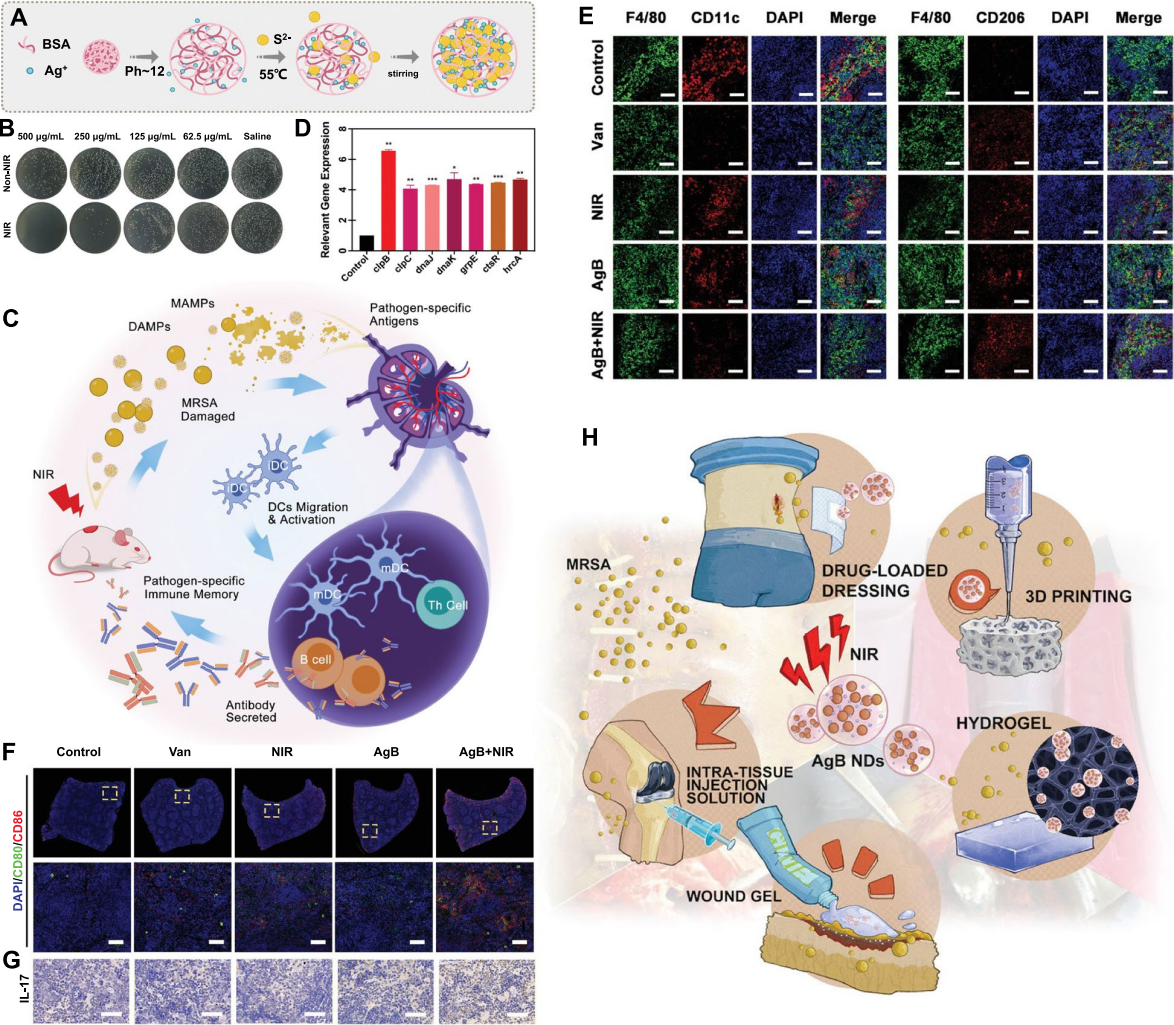
Multifunctional photosensitizer nanodots eliciting immunogenicity. **A** AgB ND preparation. **B** Antibacterial effect of AgB NDs. **C** Immunoregulation mechanism of AgB NDs. **D** the relevant expression level of heat shock stress and antioxidative stress-related genes. **E** Immunofluorescence staining of the macrophage subtype (M1-type marker, F4/80/CD11c; M2-type marker, F4/80/CD206). **F** mDC fluorescence staining in the spleen. **G** Analysis of IL-17 in infected tissue. **H** Schematic diagram showing the potential clinical application of AgB NDs [49]. Copyright © 2022 Wiley‐VCH GmbH

Due to the prevalence of drug-resistant bacteria, therapy for postoperative infections with high morbidity is extremely difficult. Motived by the superior performance of thermoinduced immunogenic cell death (ICD) in oncotherapy, Yue et al*.* constructed AgB nanodots (NDs) for multimodal synergistic antimicrobial therapy and immune regulation (Fig. 10A) by using Ag^+^ and bovine serum albumin raw materials [49]. The ICD of methicillin-resistant *Staphylococcus aureus* (MRSA) generated from AgB ND-mediated antibacterial therapy based on PDT, PTT, and Ag ion release (Fig. 10B) could then expedite DC maturation, mediate macrophage phenotypic differentiation, stimulate T cells, and endow the immune system with immunological memory to resist the next invasion of MRSA (Fig. 10C). Specifically, AgB nanodots serve as immunostimulants by improving host immunogenicity. Meanwhile, after AgB administration, the host could build adaptive immune memory by improving the expression level of heat shock stress (*e.g., clpBC*, *ctsR*, *dnaJK*, and *grpE*) and antioxidative stress-related (e.g., *dps*) genes (Fig. 10D). This stimulus enhanced the antimicrobial properties of the immune system by promoting M1-type macrophage differentiation, inducing the maturation of DCs, stimulating T cells, and eliciting immune memory B-cell production for the removal of secondary infectious bacteria (Fig. 10E-G). The above AgB nanodot strategy provides a novel systematic immunotherapeutic method to manage persistent infections, thereby significantly decreasing the recurrence rate after the primary infection is cured. This nanoplatform could also guide doctors focused on seeking promising applications in the clinic to improve the therapeutic effect of infectious diseases caused by drug-resistant pathogens (Fig. 10H).

### Sonodynamic antibacterial nanoplatforms for synergistic immune regulation

**Fig. 11:**
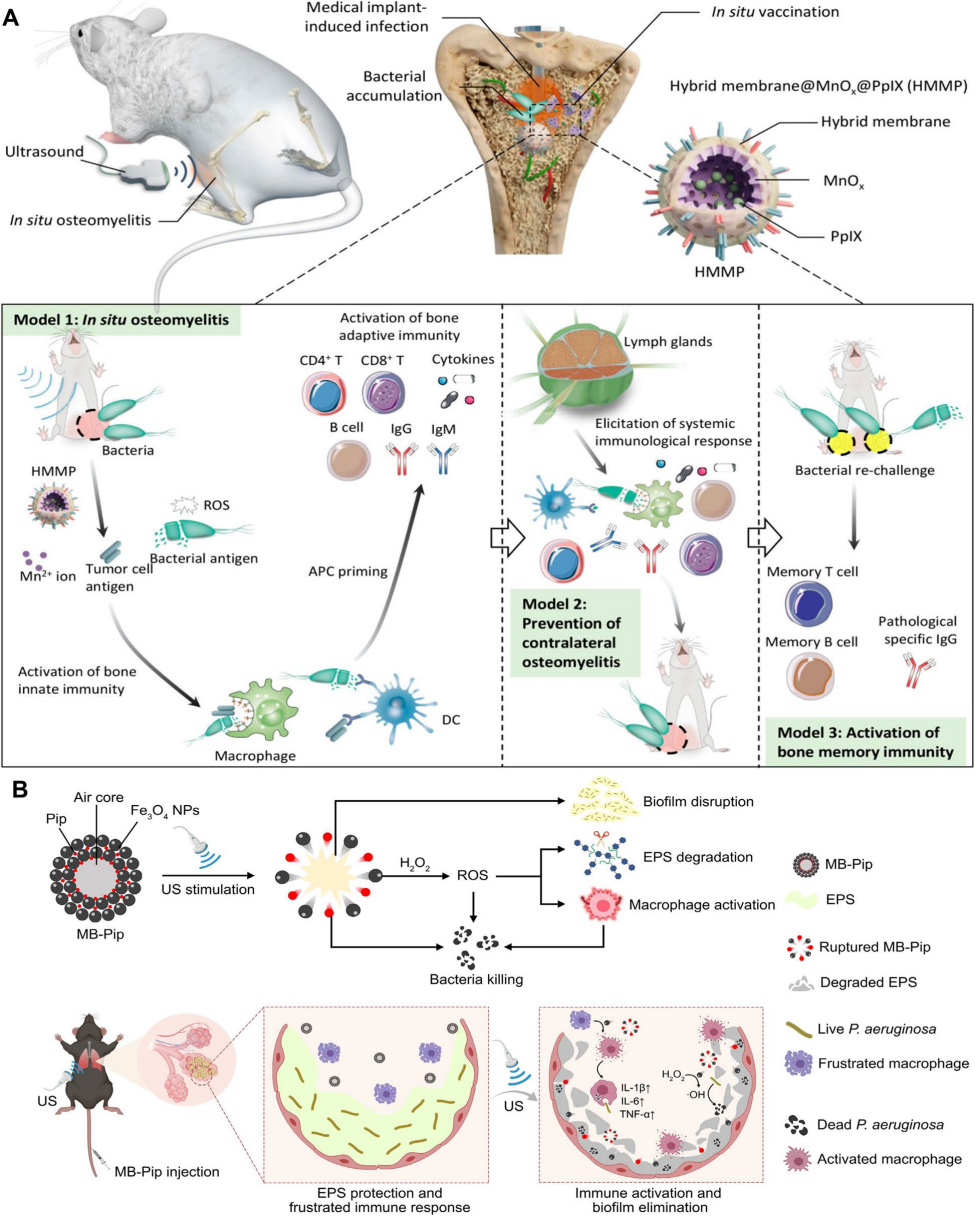
Antibacterial sonodynamic nanoplatforms. **A** HMMP nanovaccine for the treatment of osteomyelitis. Model 1: nanovaccines activate explosive antigen release, prompting APC priming and eliciting immunoantimicrobial therapy under US stimulation; Model 2: nanovaccines can activate systemic immunological reactions to remove the bacteria; Model 3: nanovaccines confer recipient with immune memory for combating bacterial rechallenge [97]. Copyright©2022, American Chemical Society. **B** Schematic illustration of US-responsive catalytic MBs for treating chronic lung infections [98]. Copyright©2023, Xiu et al*.* American Association for the Advancement of Science

Unlike aPDT, antibacterial sonodynamic therapy (aSDT) employs low-frequency ultrasound to activate sonosensitizers to generate ROS for the elimination of pathogens [35, 96]. Compared with aPDT, aSDT holds wider clinical application prospects because ultrasound possesses a deeper tissue penetration depth [35]. Recent studies have confirmed that aSDT can kill deep-seated bacteria and induce corresponding immune responses. For example, hollow MnOx preloaded with PpIX was encapsulated with a hybrid biomembrane that originated from 4T1 tumor cells and RAW264.7 macrophage cells [97]. The resulting nanomedicine (HMMP) can induce vaccine generation in situ by an aSDT-mediated antibacterial strategy (Fig. 11A). The cell membrane coating at MnOx acts as an immunoadjuvant and can attract and revitalize antigen-presenting cells at the pathological site where pathogen-related antigens are produced after the pathogens are inactivated by ROS; thus, the adaptive antimicrobial immune reaction is activated and magnified. In the management of bacteria‐infected osteomyelitis, HMMP not only eliminated the pathogen but also induced a powerful systemic antibacterial immune response. Moreover, patients with long-lasting immune memory could avoid infection recurrence after administration of HMMP. This work provides a pathway for the development of personalized antimicrobial immunotherapy.

However, some pathogenic bacteria have evolved strategies to resist clearance by the host’s immune system [64, 99]. For example, the toxin secreted by *Bacillus anthracis* can enter immune cells and subvert immune signaling pathways, further resulting in immunosuppression and even immune evasion [64]. Therefore, neutralizing bacterial toxins is a promising strategy to safeguard the host immune system. For instance, Liu and Wu et al. utilized a cell membrane-modified sonodynamic agent to neutralize the toxins produced by pathogens, further achieving synergistic sonodynamic immunotherapy [100, 101]. As a representative example, Wu et al. synthesized an erythrocyte membrane-functionalized sonodynamic agent (HNTM-Pt@Au) for the treatment of *Staphylococcus aureus*-infected osteomyelitis [101]. Pt atoms were introduced into the zirconium porphyrin metal–organic framework (HNTM) to improve the sonocatalytic activity of HNTM. Ultrasonic-triggered gold nanorods (Au NRs) were modified on the outer layer of HNTM-Pt to boost the aSDT outcome by enhancing cavitation generation and electron transfer ability. Finally, the erythrocyte membrane was modified onto the surface of HNTM-Pt@Au to endow it with toxin neutralization performance. Using the HNTM-Pt@Au therapeutic agent, *Staphylococcus aureus*-infected osteomyelitis was successfully cured, which confirms that the combination of toxin neutralization and aSDT is an alternative approach for the management of osteomyelitis. In addition, some sonodynamic agents that promote immune cell proliferation and migration have been developed for the treatment of bacteria-infected wounds [102]. Xiu et al*.* designed self-assembled microbubbles composed of Fe_3_O_4_ nanoparticles (NPs) loaded with piperacillin (MB-Pip) to eliminate biofilms and activate the immune response to treat chronic lung infections [98]. MB-Pip can promote physical disruption of Pseudomonas aeruginosa biofilms and penetration of Pip, and the released Fe_3_O_4_ NPs with peroxidase-like catalytic activity can catalyze H_2_O_2_ to generate hydroxyl radicals (•OH) and chemically degrade the biofilm matrix (Fig. 11B). Moreover, MB-Pip can induce macrophage M1 polarization, which improved the aberrant state of the host inflammatory response due to the virulence factors secreted from bacteria.

### Microwave thermal-dynamic antibacterial nanoplatforms for synergistic immune regulation

**Fig. 12:**
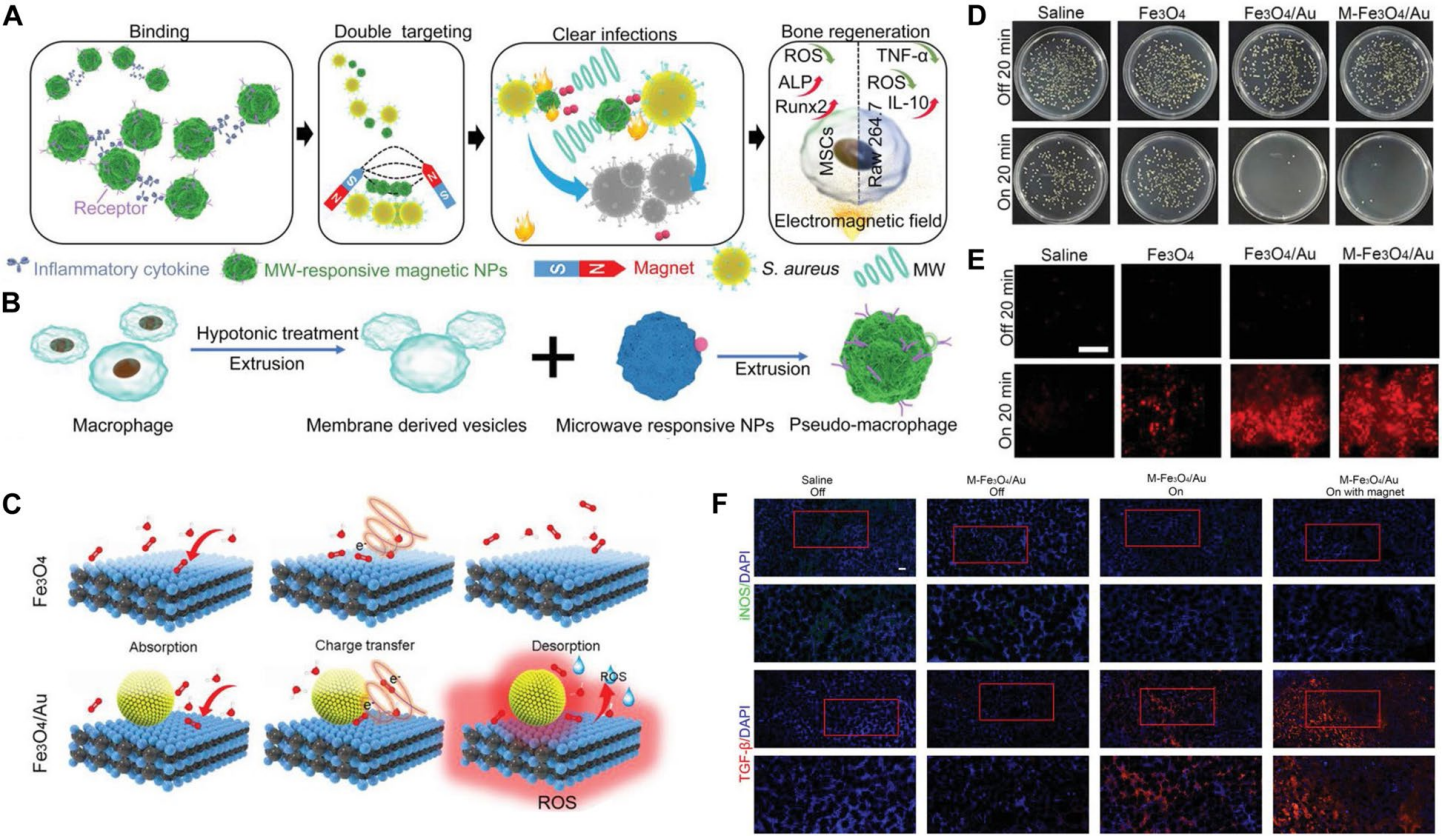
M-Fe_3_O_4_/Au NPs for microwave-triggered thermal and dynamic antibacterial therapy and immunoregulation. **A** Schematic diagram of M-Fe_3_O_4_/Au NPs for the treatment of osteomyelitis. **B** The fabrication process of M-Fe_3_O_4_/Au NPs. **C** Microwave-triggered catalytic process of Fe_3_O_4_ and M-Fe_3_O_4_/Au NPs under microwave stimulation. **D** LB plates show the antibacterial activity of microwave-triggered thermal and dynamic antibacterial therapy. **E** Membrane integrity analysis using PI dye. **F** Tissue immunofluorescence analysis (M1 marker, iNOS, green; M2 marker, TGF-β, red) [103]. Copyright©2021 Wiley‐VCH GmbH

Microwave-mediated antibacterial therapy has a bright future because microwaves have deep tissue penetration and will not induce significant damage to the perienchyma. However, microwave-activated agents with a single thermal effect cannot eradicate pathogens effectively. Therefore, the combination of microwave thermal therapy and other antibacterial methods (*e.g.*, microwave dynamic therapy) is indispensable. For example, Wu et al. synthesized macrophage membrane-coated ferroferric oxide/gold composites (M-Fe_3_O_4_/Au NPs) for microwave-triggered thermal/dynamic therapy owing to the existence of gap plasmons and electromagnetic hotspots after gold was introduced onto Fe_3_O_4_ NPs (Fig. 12A-E) [103]. With the assistance of microwaves, the macrophages were polarized toward M2-type macrophages by increasing the expression of IL 10 and decreasing the expression of tumor necrosis factor α (TNF-α, an M1 marker); meanwhile, the deep tissue pathogens could be eradicated effectively through the combination of M-Fe_3_O_4_/Au NPs and microwaves. In addition, M-Fe_3_O_4_/Au NP-treated macrophages reduced the secretion of inflammatory cytokines and consequently downregulated ROS generation in bone marrow mesenchymal stem cells, facilitating osteogenic differentiation (Fig. 12F). This study first confirms that the combination of microwave-induced therapy and immune regulation can be used for the treatment of bacteria-induced osteomyelitis.

### Magnetic hyperthermia antibacterial nanoplatforms for synergistic immune regulation

**Fig. 13:**
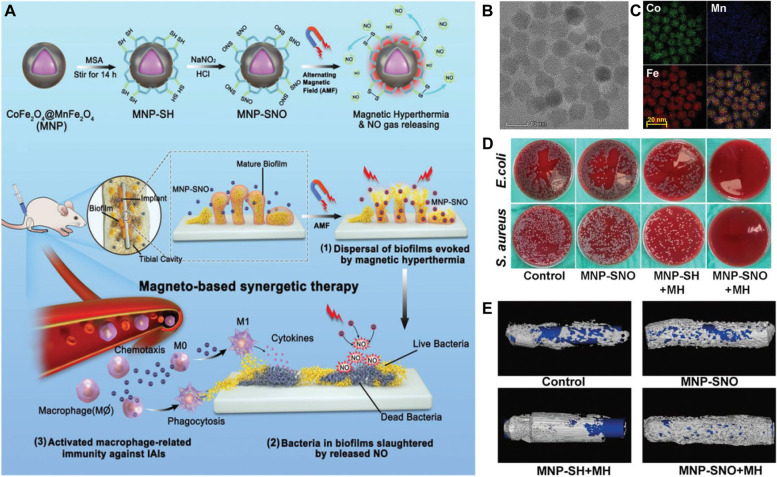
Magneto-based synergetic therapy for implant-associated infections. **A** Schematic illustration of the synthesis of MNP-SNOs and their magneto-based synergetic therapy. **B** TEM and **C** EDS mapping images of the magneto-based nanoplatform. **D** Typical photos of E. coli and S. aureus biofilms in different groups. **E** 3D reconstructions of new bones and implants in different groups [105]. Copyright © 2021 The Wang et al*.* Advanced Science published by Wiley–VCH GmbH

Nitric oxide (NO), as an antibiofilm therapeutic agent, can penetrate the inside of bacterial biofilms and kill bacteria within biofilms [104]. Among the NO donors, S-nitrosothiols (RSNO) are a thermotriggered NO generator that can be activated to release NO to attack the pathogen under magnetic hyperthermia. Therefore, the integration of hyperthermial therapy and NO gas therapy can not only destroy the structure of bacteria and biofilms but also implement on-demand release for precision therapy. Although the combination of hyperthermia and NO gas therapy is capable of eliminating the mature biofilm, the few remaining bacteria may still cause a recurrence of the infection due to the innate immune damage caused by bacterial biofilms, especially in implant‐associated biofilm infections. To overcome this issue, Shi et al*.* developed SNO-functionalized CoFe_2_O_4_@MnFe_2_O_4_ nanoparticles (MNPs‐SNO) to fight against implant‐associated infectious diseases (Fig. 13A-C) [105]. Once exposed to an alternating magnetic field, MNP NPs could convert electromagnetic energy into heat to induce biofilm dispersal by magnetic hyperthermia therapy, further enhancing the permeability of MNP‐SNO in the biofilm and treating implant infection (Fig. 13D, E). Subsequently, the hyperthermia generated from MNP‐SNO induces the release of NO to inactivate the pathogen within the biofilm. Moreover, immunosuppression in biofilm-infected sites can be reversed after the administration of MNP‐SNOs. In rodent models, the authors found that more macrophages were recruited and converted into M1-type macrophages to trigger the immune system to avoid the recurrence of infection. This work offers an alternative strategy to control deep implant‐associated biofilm infections by using magnetothermal/NO gas/immunotherapy.

## Endogenous microenvironment-responsive nanoplatforms

### Nanoplatform-mediated gas therapy regulates antibacterial immunity

Given the potential bacterial resistance of traditional antibiotics, gas therapy has been proved to be an alternative solution for antibacterial applications. As a result, many efforts have been made to design gas-releasing nanoplatforms that are response to the infected microenvironment for antibacterial treatments [106]. Gas molecules such as NO, sulfur dioxide (SO_2_), carbon monoxide (CO), and hydrogen sulfide (H_2_S) play vital roles in physiological processes. In addition, these gases can not only kill cancer cells or pathogens at high concentrations but also possess immunomodulatory activity [107]. Biofilm formation is the major reason for the occurrence of chronic infected wounds. Due to the presence of a biofilm barrier, the therapeutic agent cannot effectively penetrate the biofilm, and subsequently, the bacteria cannot be effectively removed, further resulting in a chronic inflammatory state. To improve the therapeutic effect of biofilm-infected chronic disease, an NO-propelled nanorobot was constructed by surface locally modifying Fe_3_O_4_ NPs with polydopamine biomacromolecule, where polymyxin B (PMB) was subsequently modified to the surface of the particle via polydopamine coating, and the NO donors were reacted with -SH groups to obtain IO@PMB-SNO NPs [108]. A high GSH content in biofilms can induce NO generation and propel IO@PMB-SNO NP nanomaterials to the deep biofilm layers. Simultaneously, the produced NO can kill bacteria within biofilms, and the bacterial residue can be removed under a magnetic field because PMB tend to bind with bacteria. In vivo*,* the removal of the biofilm matrix and bacterial fragments by magnetic separation technology could significantly relieve chronic inflammatory reactions, and the expression of proinflammatory cytokines (e.g., IL-6 and TNF-α) was obviously decreased. After in vivo administration, IO@PMB-SNO NMs could promote chronic infected wounds by modulating wound inflammatory reactions and enhancing biofilm clearance via nanorobots.

In addition to the excessive inflammatory response and high reactive oxygen levels, a detrimental microenvironment, such as hyperglycemia, hypoxia, and low NO levels, exists in diabetic wounds, which will impair wound healing [109, 110]. Moreover, the moist and hyperglycemic wound environment is beneficial for bacterial reproduction and even biofilm formation, resulting in the development of chronic infected wounds. To confront these challenges, Gao et al. employed hyperbranched poly-L-lysine-functionalized manganese dioxide (MnO_2_) nanosheets and poly (PEGMA-co-GMA-co-AAm) (PPGA) crosslinkers to construct an HMP hydrogel to deliver pravastatin sodium to enhance the synthesis of NO in infected wounds [111]. The MnO_2_ nanozyme embedded in the HMP hydrogel with a higher stability can reduce high levels of ROS at the site of infection by converting ROS into oxygen. The polylysine component in the HMP hydrogel could effectively kill gram-positive and gram-negative bacteria. Thus, the degree of inflammatory reaction was relieved, and more M2-type macrophages were induced to enhance chronic infected wound healing. Moreover, by using the catalase-like catalytic reaction of MnO_2_ nanozyme, the oxygen generated from the high concentration of H_2_O_2_ in the infected tissue and NO production enhanced by pravastatin could further accelerate wound healing. The results of the wound treatment experiment indicated that the HMP hydrogel could promote angiogenesis, enhance the generation of TGF-β, and stimulate collagen formation. Therefore, the HMP hydrogel can be used as a therapeutic agent for accelerating the healing of chronically infected wounds and even scarless wounds.

**Fig. 14:**
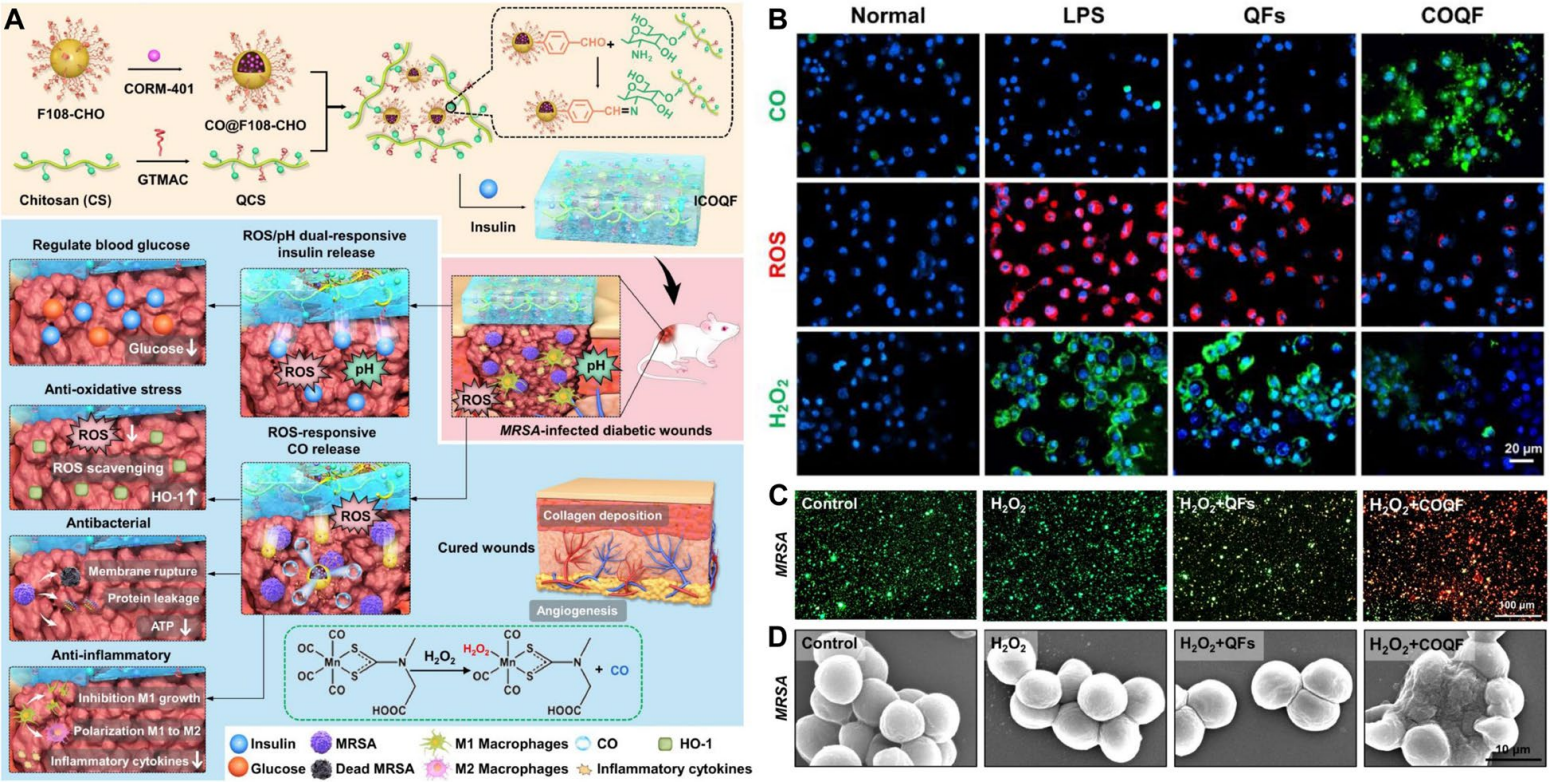
CO gas therapy-based Nanoplatform. **A** Schematic illustration of the preparation of the ROS-responsive antibacterial system (ICOQF) and its antihyperglycemic, antibacterial, antioxidative, and anti-inflammatory mechanisms in the therapy of diabetic infected wounds. **B** Fluorescence imaging of intracellular CO, ROS, and H_2_O_2_. **C** Live/dead staining and **D** SEM images of MRSA in different treatment groups [112]. Copyright©2022 Acta Materialia Inc., Elsevier Ltd

CO with anti-inflammatory properties has been widely investigated. Research results indicate that CO is involved in the regulation of the activation of heme oxygenase (HO-1), the mitogen-activated protein kinase (MAPK) pathway and so on. For example, Cai et al. developed an ROS-responsive antibacterial system for the on-demand release of CO (Fig. 14A, B) [112]. In this system, an ROS-triggered CO donor (CORM-401) was embedded in benzaldehyde-terminated F108 nanoparticles and then mixed with quaternized chitosan (QCS) to fabricate an ICOQF hydrogel. Then, insulin is added to the gel precursor to endow the ICOQF hydrogel with glycemic regulatory performance. Using the ICOQF hydrogel, the pathogens in the wound could be eradicated effectively (Fig. 14C, D), and CO gas also suppressed macrophage cell reproduction and was beneficial for macrophages from the M1 to the M2 type. transformation of M1-type macrophages into M2-type macrophages, further remodeling the detrimental diabetic infected wound and enhancing diabetic wound closure.

H_2_S gas can reverse drug resistance, thereby increasing the sensitivity of drug-resistant bacteria to antibiotics [113, 114]. Recent work indicated that H_2_S can also increase the sensitivity of bacteria to photothermal treatment. For example, Zhu et al. developed a pH/GSH dual-responsive antibacterial agent (MSDG) by coating Prussian blue vehicles with a metal–organic framework (MOF) [26]. Upon exposure to the acidic biofilm microenvironment, the acidity-sensitive MOF protective layer was destroyed to release the preloaded diallyl trisulfide. Then, the diallyl trisulfide reacts with glutathione (GSH) to generate H_2_S gas. Upon irradiation with an 808 nm laser, MSDG-based H_2_S-sensitized photothermal therapy effectively removed bacterial biofilms by heat-inactivated pathogens and H_2_S-mediated extracellular DNA removal. The H_2_S gas from the reaction of diallyl trisulfide and GSH enriched in the biofilm could remodel the immune microenvironment by inducing the production of M2-type macrophages. Then, M2-type macrophages secrete regeneration-associated cytokines to promote wound repair. From the results of immunohistochemistry analysis, high α-SMA and CD31 protein contents were observed, which are myofibroblast and endotheliocyte markers, respectively. This work constructed a biofilm environment-responsive antibacterial platform for combating intractable implant-associated infections by combining H_2_S gas/photothermal/immunotherapy.

### Chemodynamic antibacterial nanoplatforms for synergistic immune regulation

**Fig. 15:**
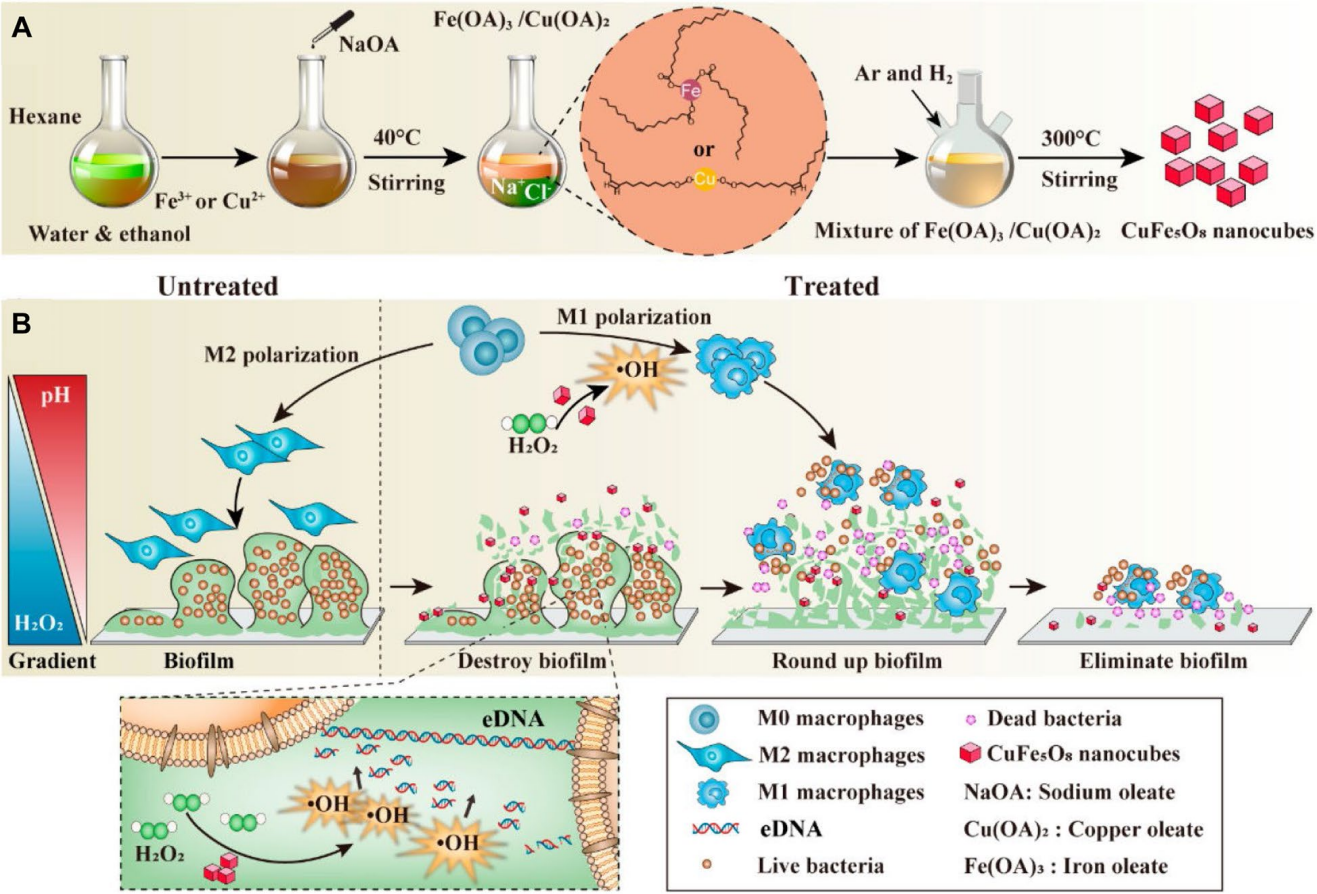
Chemodynamic therapy regulates macrophages for implant-related infections. **A** Schematic illustration demonstrating the synthetic processes. **B** Antibiofilm and immunoregulation mechanisms of CuFe_5_O_8_ NCs [119]. Copyright©2020, American Chemical Society

Antibacterial chemodynamic therapy (aCDT), a novel antibacterial strategy that executes antibacterial activity through the degradation of hydrogen peroxide (H_2_O_2_) to produce toxic hydroxyl radicals (•OH) by Fenton-like activity, has been widely studied [8, 115, 116]. By using aCDT, a high concentration of H_2_O_2_ in biofilm-infected tissue can be converted into •OH for sterilization with a negligible effect on peripheral normal tissue [117]. Recent research results claim that a high concentration of ROS can destroy biological macromolecules and lead to bacterial damage or even death, while a low concentration of ROS can trigger the secretion of proinflammatory cytokines in immune cells, thus reinforcing the inflammatory reaction and enhancing the performance of immune cells to remove pathogens [118]. Thus, the existence of a H_2_O_2_ concentration gradient in the biofilm can serve as an environmental stimulus for designing a synergistic therapeutic system for aCDT and immunotherapy. For example, Zhang et al*.* employed a spatial adaptive chemodynamic therapeutic approach to control implant biofilm-related infection by using a pH/H_2_O_2_-responsive Fenton-like nanocatalytic agent (CuFe_5_O_8_ nanocubes) (Fig. 15A) [119]. Due to the existence of a high H_2_O_2_ concentration and low pH microenvironment in the biofilm, abundant •OH was generated to destroy the structural integrity of biofilms by disrupting the main component of biofilms, extracellular DNA. Nevertheless, there is a high pH and low H_2_O_2_ concentration at the edge of the biofilm, and a small amount of •OH generated from the Fenton-like reaction could effectively rejuvenate innate immunity by driving native macrophage (M0) polarization toward proinflammatory macrophages (M1-type) (Fig. 15B). The combination of aCDT and immunotherapy achieved good therapeutic effects in implant biofilm-related infection management *ex/*in vivo because macrophages could eliminate biofilm debris, which offers an alternative approach to combat implant biofilm-related infection.

## Conclusions and future perspectives

### Advantages of nanotherapeutics with immune regulation ability

The emergence of superbugs poses a serious challenge to the current health care system. Therefore, there is a great need to develop effective, safe and low-resistance antimicrobial strategies. Antibacterial immunotherapy mainly depends on the immune system to obliterate the pathogenic bacteria, thereby avoiding the occurrence and recurrence of bacterial infection, showing unique superiority in the treatment of bacterial infection. Moreover, long-term immune memory helps the body efficiently remove infectious pathogens. However, the underlying adverse effects of traditional immune adjuvants limit their clinical application. First, the targets of traditional immune adjuvants are not specific. For instance, traditional immunopotentiators not only strengthen the body’s immune reaction but also cause tissue damage [120, 121]. Second, the expenditure of antibody production is huge. Furthermore, there are currently no clinically approved antimicrobial monoclonal antibody formulations due to poor antibody specificity and short circulating half-life in vivo [99]. Furthermore, the direct use of immune-regulated antibodies may induce side effects such as antigen–antibody complex disease [20]. Third, some immune checkpoint blockades not only strengthen the immune system to eliminate pathogens but also magnify collateral tissue damage [17]. To tackle these challenges, nanomaterial-based vaccines with satisfactory biocompatibility, long-term body circulation, and superior targeting performance can serve as multifunctional nanoplatforms to combine different therapeutic strategies and achieve the effect of combination therapy. With the development of nanotechnology, smart antibacterial platforms with immunomodulatory properties have been tailored to specifically enrich and release therapeutic agents on demand at the site of infection, which can utilize the autoimmune system to enhance the pathogen removal effect and minimize the dosage of medication and the adverse effect on normal tissue. In addition, nanotechnologies destroy complex biofilm structures and eradicate mature bacterial biofilms are involved in improving the local immune microenvironment of bacterial infections. Nanoplatforms can also enhance the ICD response for immunological treatments for persistent infections. The ICD of bacteria, serves as an exogenous stress that stimulates the immune system and elicits immunogenicity, which can induce immune responses, including increased transmigration of lymphocytes into tissues. Thus, nanotherapeutic strategies can effectively promote the maturation of DCs, regulate the polarization of macrophages, and activate T cells while inducing postoperative active long-term immune monitoring and addressing the challenge of recurring infections.

### The issues of optimizing nanoplatforms with immunoregulatory functions need considered

#### Toxicity, targeting, and stability evaluation

The biocompatibility, targetability and stability of nanotherapeutic agents should still be systematically evaluated. Previous studies confirmed that some unmetabolizable nanomaterials, especially inorganic nanomaterials, can be enriched in vivo for a long time, resulting in the development of chronic inflammation. Ideal antibacterial agents need to specifically accumulate in the infected site and then release bioactive drugs on demand without premature leakage, which can enhance the therapeutic outcome while minimizing side effects. The infection-specific microenvironment, such as low pH, high local temperature, and high toxin and hydrolase levels in infected tissue, could serve as triggers for targeted therapy. In addition, external stimuli, such as light, ultrasound, and magnetism, can also be used for the design of responsive nanotherapeutic agents. More importantly, the stability of nanotherapeutic agents in the biological environment is a prerequisite for infection targeting therapy. Therefore, the long-term stability of nanotherapeutic agents should be evaluated before clinical use.

#### Adequate preclinical test

More preclinical studies need to be implemented. Although the developed nanotherapeutic agent with immunomodulatory properties can directly inactivate infectious pathogenic bacteria or modulate the immune system to remove the bacteria in vitro or in a mouse model, few assessments have been performed on large animals, let alone people. Therefore, the pharmacodynamics, pharmacokinetics, and toxicity of nanotherapeutics in the body should be systematically investigated before clinical translation.

#### Evaluation of multimodal antibacterial activities and operational principles

Multimodal antibacterial therapy has become routine, and the combined therapeutic effect should be improved. In general, multimodal nanotherapeutic formulas possess higher antibacterial performance and immune regulation abilities while minimizing the shortcomings of unimodal therapy. In addition, preventing the recurrence of infectious diseases is very important to improve the quality of life of patients. Many studies have confirmed that nanoplatforms inducing immunological memory in the adaptive immune system can effectively avoid the recurrence of bacterial infection. Therefore, the application of nanoplatforms to maintain long-term anti-infection performance and reduce the burden of pathogens in infected tissues requires systematic analysis and comprehensive evaluation of their efficacy.

#### Investigation of the immune escape mechanism

It is noteworthy that some infectious pathogens have evolved strategies to escape immune system clearance. The immune escape mechanism is extremely complex and unclear. Therefore, using nanotechnology to decipher the immune escape mechanism and block the immune escape process can greatly enhance the immune system to eliminate antimicrobial performance.

#### Overcoming bacterial biofilm fortress

Although different therapeutic approaches, such as PTT, PDT, SDT, gene therapy, chemotherapy, and gas therapy, can be integrated into one therapeutic nanoplatform, they can induce ICD of bacterial immunogenic cell death, further creating a strong immunogenic infection microenvironment with the release of DAMPs. However, the effectiveness of nanoplatform-mediated antibacterial treatments is greatly constrained by bacterial biofilms. The bacteria within the biofilm cannot be eliminated completely because the biofilm matrix barrier hinders drug/immune cell penetration into the biofilm. Therefore, it is necessary to develop more effective nanoplatforms with high biofilm permeability for bacterial infection treatment.

## Data Availability

Not applicable because this is a review article and no data were newly generated.
